# Active Micro-Nano-Collaborative Bioelectronic Device for Advanced Electrophysiological Recording

**DOI:** 10.1007/s40820-024-01336-1

**Published:** 2024-02-27

**Authors:** Yuting Xiang, Keda Shi, Ying Li, Jiajin Xue, Zhicheng Tong, Huiming Li, Zhongjun Li, Chong Teng, Jiaru Fang, Ning Hu

**Affiliations:** 1 Department of Chemistry, Zhejiang-Israel Joint Laboratory of Self-Assembling Functional Materials, ZJU-Hangzhou Global Scientific and Technological Innovation Center, Zhejiang University, Hangzhou, 310058 People’s Republic of China; 2 School of Basic Medical Sciences, Zhejiang Chinese Medical University, Hangzhou, 310053 People’s Republic of China; 3 General Surgery Department, Children’s Hospital, Zhejiang University School of Medicine, National Clinical Research Center for Children’s Health, Hangzhou, 310052 People’s Republic of China; 4 Department of Lung Transplantation and General Thoracic Surgery, The First Affiliated Hospital, Zhejiang University School of Medicine, Hangzhou, 310003 People’s Republic of China; 5 Department of Orthopedics, The Fourth Affiliated Hospital, Zhejiang University School of Medicine, Yiwu, 322005 People’s Republic of China; 6 School of Electronics and Information Technology, Sun Yat-Sen University, Guangzhou, 510006 People’s Republic of China; 7 Department of Obstetrics and Gynecology, The Tenth Affiliated Hospital, Southern Medical University, Dongguan, 523059 People’s Republic of China; 8 Dongguan Key Laboratory of Major Diseases in Obstetrics and Gynecology, Dongguan, 523059 People’s Republic of China

**Keywords:** Active micro/nano collaborative bioelectronic device, Three-dimensional active nano-transistor, Planar active micro-transistor, Electrophysiology

## Abstract

The development of precise and sensitive electrophysiological recording platforms holds the utmost importance for research in the fields of cardiology and neuroscience. In recent years, active micro/nano-bioelectronic devices have undergone significant advancements, thereby facilitating the study of electrophysiology. The distinctive configuration and exceptional functionality of these active micro-nano-collaborative bioelectronic devices offer the potential for the recording of high-fidelity action potential signals on a large scale. In this paper, we review three-dimensional active nano-transistors and planar active micro-transistors in terms of their applications in electro-excitable cells, focusing on the evaluation of the effects of active micro/nano-bioelectronic devices on electrophysiological signals. Looking forward to the possibilities, challenges, and wide prospects of active micro-nano-devices, we expect to advance their progress to satisfy the demands of theoretical investigations and medical implementations within the domains of cardiology and neuroscience research.

## Highlights

- The factors affecting electrophysiological recordings from active micro-nano-collaborative bioelectronic devices were discussed in terms of principle and fabrication.
- An overview of the applications of active micro-nano-collaborative bioelectronic devices in cardiomyocytes and neurons was further presented.
- The challenges faced by active micro-nano-collaborative bioelectronic devices in intracellular electrophysiological studies and their prospective biomedical applications are discussed.

## Graphical abstract

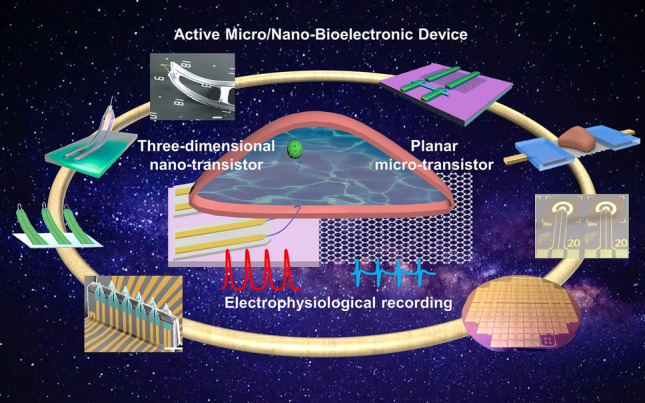

## Introduction

Many important physiological activities in the human body, such as excitation–contraction coupling in the cardiac muscle and excitatory transmission in the brain, are inseparable from cellular electrophysiology. Electrophysiology has been used to clarify and regulate the activities of electro-excitable cells [1–6]. Cardiovascular diseases remain a major cause of morbidity and mortality on a global scale [7, 8]. To prevent and treat cardiovascular diseases by examining their pathogenesis, it is crucial to create a dependable platform that facilitates the monitoring and analysis of myocardial or neuronal electrophysiology at the cellular level [9–14]. Therefore, cardiomyocytes and neurons have received a lot of attention from researchers as typical electrically excited cells (cardiomyocytes, somatic cells, neurons, and glia cells [2, 4–6, 15]) in electrophysiological research of the heart and brain. Research in this field is predominantly conducted through the utilization of established techniques for recording high-quality electrophysiological signals in individual cells or cell networks. Under ideal conditions, highly accurate electrophysiological signal recordings can be obtained by performing intracellular sensing [16, 17].

At present, diverse forms of patch-clamp have established themselves as the benchmark for capturing electrophysiological signals. The patch-clamp technique, a microelectrode technique that employs a clamped voltage or current to record ion channels' electrical activity on cell membranes, enables the acquisition of high signal-to-noise ratio (SNR) electrophysiological signals. Nevertheless, the execution of patch-clamp on multiple cells concurrently poses difficulties. Furthermore, the irreparable harm to the cells and the intricate manipulation process do not facilitate the recording of multiple cells for a long period simultaneously [18–24]. Voltage-sensitive dye-based methods can record multiple cells simultaneously but are plagued by issues, such as low SNR, phototoxicity, and limited temporal resolution [25–30]. Therefore, several scalable methods for electrophysiological signal recording have been explored, including passive electrodes [31–37] and active micro/nano-bioelectronic devices [38–46]. Microelectrode arrays (MEAs) [31, 47–52] are passive electrodes that are commonly produced on insulating substrates. These electrodes receive electrical signals from cells and subsequently transmit them to external amplifiers through leads that are coated with passivation layers. The implementation of millimeter-scale microelectrode arrays (MEAs) presents a feasible strategy for mitigating impedance noise and detecting subtle ion fluxes across cellular membranes. However, it is difficult to obtain subthreshold and low-amplitude cell signals due to the large impedance inherent in MEAs. In contrast, active micro-nano-transistors can overcome this limitation. Due to their ability to amplify and switch currents, the response of an organism to weak electrical signals can be amplified, resulting in higher-quality electrophysiological parameters. Advances in micro-nano-technology have facilitated the development of conventional field effect transistors (FETs) from planar Si FET [53] to silicon nanowire (SiNW) [42, 54, 55] arrays. Furthermore, to attain high-quality recordings, CMOS (complementary metal oxide semiconductor) [24, 56–58] integration has been implemented, leading to a reduction in the number of leads in the electrode layer through lead sharing. This has led to a significant rise in the number of electrodes. With their addressable nature, these devices facilitate both single-cell and cell network recordings, allowing for precise cell conditioning and mapping of electrical activity conduction. As a result, active micro-nano-transistors are well-suited for multiplexed measurements and hold great potential for sensing or scalability.

Although active micro-nano-transistors have advantages in many aspects of intracellular recording technology, their development still needs to meet the following goals [59, 60]: (1) Small size: allowing better contact with subcellular structures, minimizing invasion of cells, and recording reversible, long-term electrophysiological signals [61]. (2) High sensitivity: small-size electrodes exhibit high sensitivity and high SNR, which can sensitively reflect the details of weak signals [39]. (3) High-throughput detection: the ability to record from multiple cell-electrode interfaces enables both single-cell recording and network-scale scaling, thereby facilitating accurate localization and modulation of cells. Recent advances in nanotechnology have encouraged new devices that meet the above requirements [62, 63]. With the advancement of micro- and nanotechnology, the vast array of materials and adaptable structural design offers a plethora of possibilities for the production of nanodevices [37, 41, 53, 64]. Micro-nano-transistors, nanomaterials/micro-nanostructure-based patch-clamps, microelectrode arrays, and micro-nanostructured implantable intracortical microelectrodes are important in electrophysiological recordings [65–67]. Nanomaterials such as nanofibers, nanowires, and nanoparticles have higher sensitivity and lower noise, thus enabling the fabrication of patch clamp and microelectrode arrays with higher recording accuracy and lower noise to detect fainter electrical signals [68–72]. In addition, nanomaterials are biocompatible and can be used to develop implantable microelectrodes. In recent years, fabrication techniques for implantable intracortical microelectrodes have improved dramatically, enabling more accurate electrical signal recording from neurons while reducing damage to the brain. The recording of neuronal electrical activity in the brains of living animals (such as rats and monkeys) by implantable microelectrodes has contributed to a deeper understanding of animal behavior and brain function [73, 74]. Presently, the utilization of three-dimensional active nano-transistors and planar active micro-transistors are prevalent in the fields of cardiology and neuroscience for drug screening and disease modeling [16, 75, 76].

In this review, the advancements of active micro-nano-transistors as a detection technology are described for cellular electrophysiological recordings. Active micro-nano-collaborative bioelectronic devices based on semiconductor properties can achieve signal detection at smaller sizes in living organisms by using micro-nanotechnology, which is of great significance for studying the working mechanism of isolated cells and in vivo tissues. Compared to passive bioelectronic devices, active bioelectronic devices can amplify the response to weak electrical signals in living organisms to obtain higher-quality electrophysiological parameters due to the capability of amplifying and switching currents. First, we discuss the working principle, preparation, and performance of three-dimensional active nano-transistors and planar active micro-transistors, respectively (Sects. 2 and 3). Second, the applications of active micro-nano-transistors are summarized for cardiac and neural electrophysiology (Sect. 4). Finally, we discuss the prospective tendency of active micro-nano-transistors and their potential development in the biomedical field (Sect. 5).

## Principle of Active Micro-Nano-Transistor

### Three-Dimensional Active Nano-Transistor

Nano-transistor is a semiconductor materials-based device that controls the current by controlling the carrier density in the semiconductor. There are three electrodes: drain, source, and gate. In this case, a conductive channel made of semiconductors is formed between the drain and the source [42, 77, 78]. The gate voltage is applied to the gate electrode in conventional active nano-transistors [79]. When the gate voltage is above the threshold voltage, electrons or holes migrate toward the semiconductor-oxide interface, thus reducing the channel barrier and producing a significant tunneling effect current. On the other hand, cellular action potentials arise from the transmembrane movement of ions. Fluctuations in transmembrane electrical signals are directly caused by changes in ion concentration within the cell. Depending on the identified reference and detection locations, cells can be detected for electrophysiological signal changes. When the active nano-transistor is coupled to the cell, its processing input/output information does not require direct exchange with cellular ions, allowing interface impedance and damage to the cell to be minimized. In addition, the electrically excited cells vary the voltage applied to the gate by generating action potentials, which regulate the tunneling currents at the source (S) and drain (D). Cellular electrical signals can thus be transduced by field/potential changes on well-isolated surfaces. Therefore, the active nano-transistor can be used as a powerful tool for recording. However, the S/D electrical touchpoints for current injections and collections make the design of three-dimensional probes and their minimally invasive characteristics a great challenge [61]. To address this issue, Lieber's group designed a three-dimensional nanowire kink probe that has made a breakthrough in intracellular recording [39]. A twisted nanowire tip into the cell is defined as the sensitive region of the field-effect tube, while the S/D two sides have stayed outside the cell. Based on this, branching intracellular nanotube FETs (BIT-FETs) [41] were developed, in which branching hollow SiO_2_ nanotubes act as FET gates for three-dimensional biological probes penetrating the membrane, and the S/D ends are placed on the Si nanowires. In P-type FETs, a negative gate voltage induces an increase in conductivity due to carrier accumulation and a positive gate voltage induces a decrease in conductivity due to carrier depletion, so that the recorded conductivity is invertible concerning the potential on the gate. In addition, the cell electrophysiological signal recorded by the three-dimensional active nano-transistor is independent of the interface resistance, which records high-fidelity cell electrophysiological signal by effectively avoiding signal loss due to electrode resistance [77, 80].

**Fig. 1:**
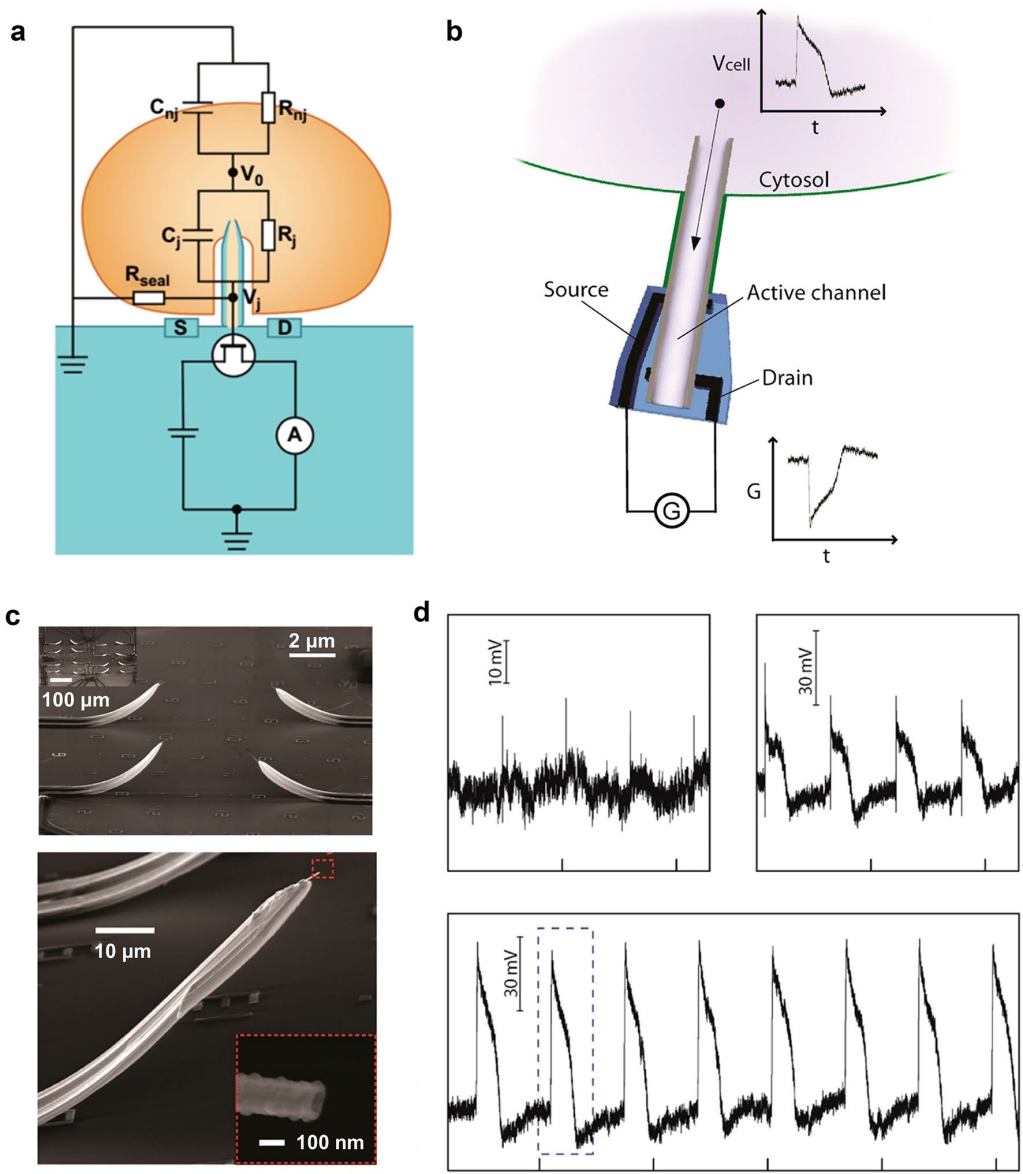
**a** Equivalent circuit model for simulating cell/nano-transistor coupling and recording electrical signals. **b** Structure and **c** SEM images of three-dimensional nano-transistor. Scale bar, 100 nm. **d** Typical extracellular and intracellular action potentials recorded after contact between nano-transistor and cardiomyocytes. Reproduced with permission from [81]. Copyright 2012, American Chemical Society

To provide a visual representation of the three-dimensional active nano-transistor record, an equivalent circuit is utilized to model the coupling between the cell and nano-transistor (Fig. 1a). The term V_0_ pertains to the action potential of a cell, while *C*_nj_ and *R*_nj_ denote non-junctional capacitance and resistance, respectively. *C*_j_ denotes junctional capacitance, which encompasses cell capacitance, nanotube capacitance, and bilayer capacitance, whereas *R*_j_ denotes junctional resistance, and *R*_seal_ pertains to seal resistance. Upon the occurrence of the action potential, *V*_0_ undergoes diffusion from the intracellular compartment. The gate voltage corresponds to the connection potential *V*_j_, while the change in *V*_j_ modulates the conductance of the nano-transistor between the source and drain electrodes. The efficacy of cell-electrode coupling in a nano-transistor can be assessed by the *V*_j_ to *V*_0_ ratio, where a higher ratio indicates superior coupling and consequently, enhanced recording proficiency [61]. Intracellular potential recording of the nano-transistor can be achieved when the nano-transistor crosses the cell membrane and enters the intracellular compartment. These models are constructed according to the commonly employed electrical model of the cell and nano-transistor. The interdependence of the nano-transistor and the cell is contingent upon the interfacial impedance that exists between the action potential and the gate, as well as the sealing resistance of the cell to the nano-transistor. Enhanced cell adhesion to the device results in a more effective seal. Figure 1b, d displays an extra- and intracellular recording of the same cardiomyocyte by the nano-transistor. For extracellular electrophysiological signal, the cell membrane remains impenetrated and therefore exhibits high impedance. For intracellular electrophysiological signal, the impedance value is close to zero as the cell cytoplasm is in contact with the gate of nano-transistor directly after penetration. The extracellular signal exhibits a different shape from the intracellular signal due to the attenuation by the membrane resistance.

### Planar Active Micro-Transistor

The most recent research trends offer new opportunities for the progression of label-free biosensors in the next generation, particularly those employing planar active micro-transistors, such as graphene micro-transistors [82]. The exceptional physical and chemical properties of graphene render it an appealing option for bioelectronic applications [83]. Graphene has good chemical stability [84] and biocompatibility [85], which is essential for integrating biological systems and applying FETs in the absence of a protective dielectric layer. Additionally, the integration of micro-transistors with flexible substrates facilitates the development of flexible devices, a critical requirement for the design of biomedical implants aimed at minimizing tissue damage and scar formation.

**Fig. 2:**
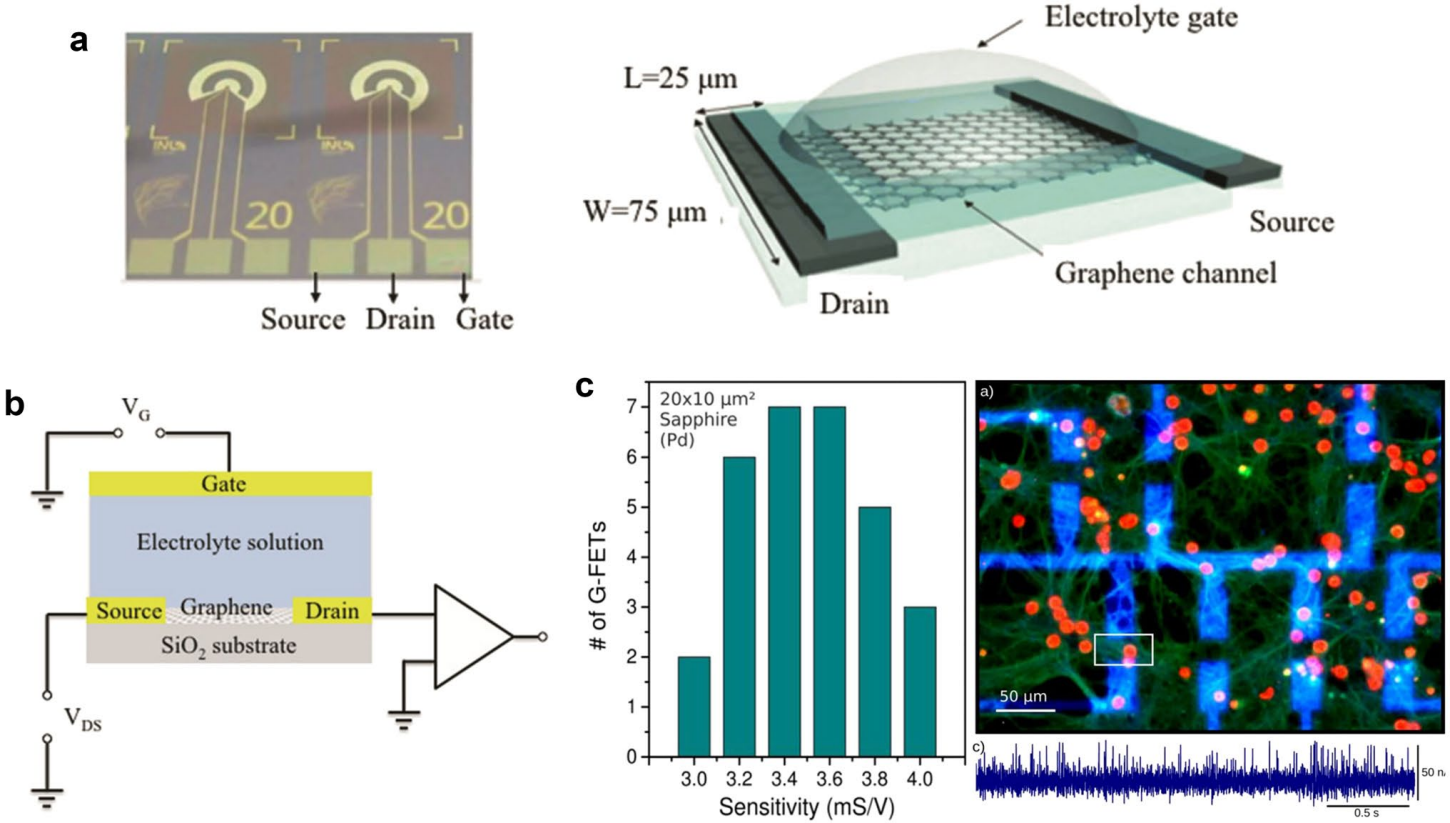
**a** Photograph of EGFET on a silicon wafer and schematic of EGFET dimensions. **b** Equivalent circuit diagram of the EGFET. Reproduced with permission from [43]. Copyright 2016, Springer International Publishing Group. **c** Sensitivity distribution ratio of 30 FETs on the same chip (*left*). Immunofluorescent maps of neurons cultured on FET and recorded electrical signal traces (*right*) [93]. Copyright 2017, Frontiers Media S.A. Publishing Group

The utilization of the field-effect mechanism in graphene nanoelectronics has facilitated the development of the initial micro-transistor, which exhibits superior performance compared to the majority of established semiconductors due to its remarkably high carrier mobility [86–88]. The ion-selective field-effect tubes (ISFET) employed in micro-transistors were initially suggested by Piet Bergveld in 1970 [89]. The sensitive region material of the micro-transistor consists of graphene, which works on the principle that electrons make the conductance of the transistor change as they pass through the graphene channels [90, 91]. The measurement between the cell and electrochemically gated graphene field-effect transistor (EGFET) is depicted in Fig. 2a, b, where the cell is positioned on the graphene surface [40, 92]. A steady bias voltage is administered to the drain and source, which are linked by a graphene conducting channel. Graphene channels in which the current is amplified and continuously monitored. Any local action potential variation caused by the cell action potential modulates the source-drain current in graphene. The constructed graphene field effect transistor has a high sensitivity (4 mS V^−1^, close to the Dirac point of *V*_LG_ < *V*_D_) and a low noise level (10^–22^ A^2^ Hz^−1^, *V*_LG_ = 0 V), which are sufficient for the measurement of extracellular electrical signals in electrically excited cells [93] **(**Fig. 2c**)**. The application of micro-transistor to high-performance label-free chemicals and biosensors has stimulated a substantial amount of experimental and theoretical research. Given the significant differences in the planar structure of graphene devices, particularly in terms of surface topography and roughness, there is a keen interest in investigating and contrasting the cellular interface of micro-transistors to evaluate the potential of graphene devices to offer distinctive functionality for bioelectronic interfaces.

## Fabrication and Performance of Active Micro-Nano-Transistor

### Three-Dimensional Active Nano-Transistor

**Fig. 3:**
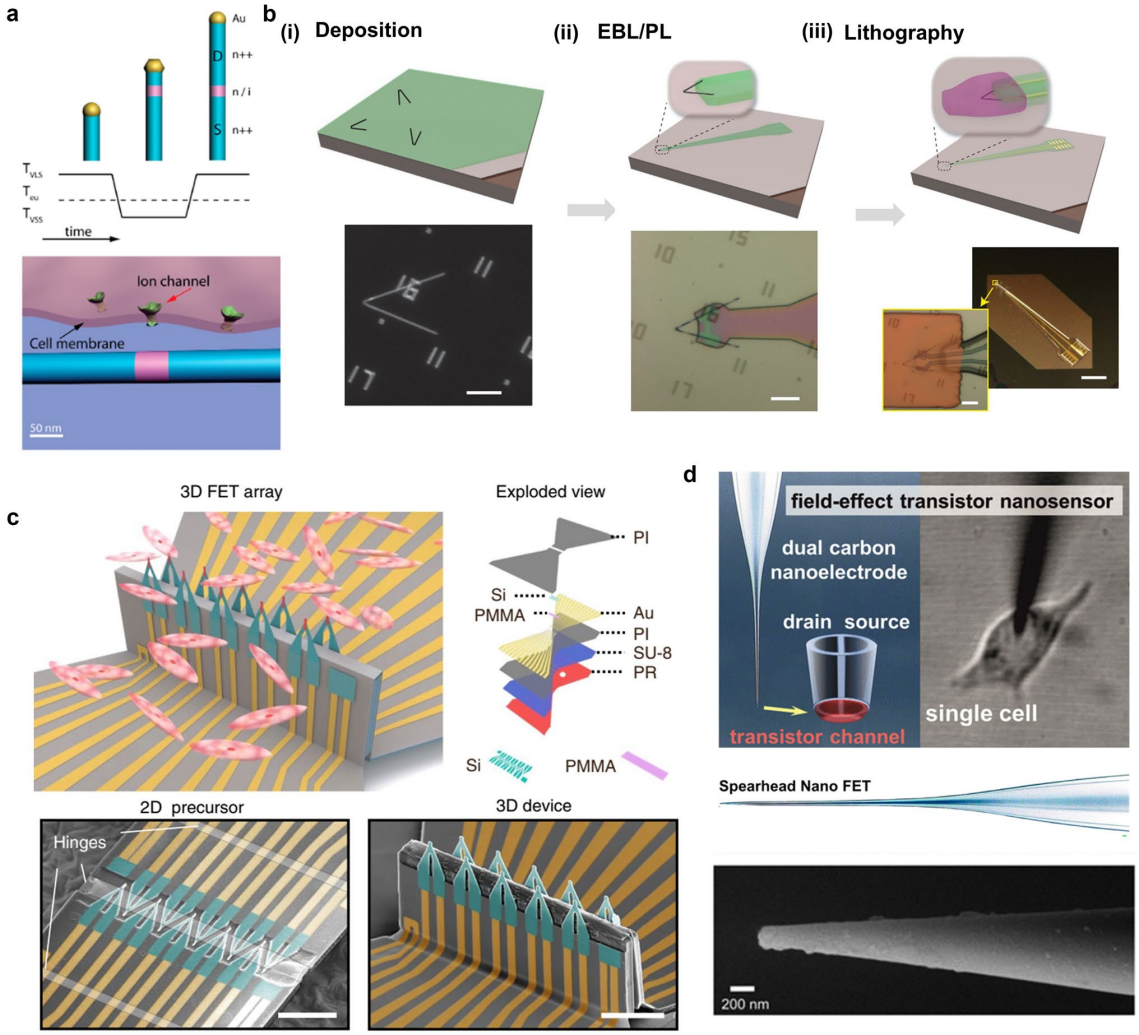
**a** Schematic representation of NW growth catalyzed by gold nanoclusters and the interface between short-channel NWFET-electrically excited cells [97]. Copyright 2012, American Chemical Society Publishing Group. **b** Nano-transistor fabrication process. FET probes were fabricated by EBL, PL, and lithography steps. Scale bar, 10 mm. Reproduced with permission from [42]. Copyright 2014, Nature Publishing Group. **c** Schematic diagram of the interface between the 10-FET array and cardiomyocytes (*left*). Multilayer structure diagram of the 10-FET array (*middle*). Pseudocolor SEM images from planar multilayer structures to three-dimensional structures, scale bars, 50 μm (*right*). Reproduced with permission from [44]. Copyright 2022, Nature Publishing Group. **d** The schematic diagram and SEM image of field-effect transistor nanosensor [109]. Copyright 2016, American Chemical Society Publishing Group

Semiconductor nanomaterial-based detectors are configured as three-dimensional active nano-transistor whose conductivity varies with the surface electric field or potential. In a standard nano-transistor, the semiconductor conductance change between the source and drain can be controlled by the gate [80, 81, 94, 95]. The conductivity dependence on the gate voltage presents the nano-transistor as a good natural candidate for biosensors because the combination of a charged material with the gate electrode produces an electric field similar to the voltage applied at the gate. The restricted sensitivity of planar devices before the earliest days prevented them from serving as chemical or biological sensors, but with the development of nanomaterials, semiconductor nanowire arrays made of silicon or other materials can also be served as nano-transistor [41, 96]. SiNW field effect transistors have good signal-to-noise ratios as biosensing platforms and can effectively couple with living cells both extracellularly and intracellularly. Although NWs have diameters of tens of nanometers, the active region of NW FET devices typically spans micrometers, confining the detection lengths and timescales of these nanodevices. The Lieber group initially reported a novel synthesis method combining gold nanocluster-catalyzed vapor–liquid-solid (VLS) and vapor–solid-solid (VSS) nanowire growth modes along the NW growth direction to produce NW devices with super-tip (< 5 nm) controllable short-channel NWs (Fig. 3a) [97]. In subsequent studies, this group demonstrated a series of nanowire field-effect transistors and branched nanotube probes. Among them, the twisted nanowire probes were fabricated by varying the vapor pressure in a VLS method reaction to obtain a 120° bent structure and separating the twisted nanowire structure with the substrate by remote electrical interconnection via electron beam lithography (Fig. 3b) [39, 98–104]. In the latest report, the group designed an ultra-small three-dimensional active nano-transistor with scalable dimensions for electrophysiological signal recording [105–108]. This nano-transistor fabrication was performed by preparing u-shaped nanowire arrays with a controlled radius of curvature from SiNW. The Si nanowire segments are then converted to metallic NiSi by depositing metal contacts through the upper Si_3_N_4_ layer and after passivation, allowing a u-shaped nanowire probe tip to produce a controlled length FET sensing element. This scalable sensor can penetrate cell membranes in a minimally invasive manner and detect low subthreshold signals within the cell.

In addition to the conventional bottom-up or top-down fabrication process, Yue Gu et al. [44] recently employed the compressive buckling technique to achieve scalable three-dimensional active nano-transistor for multi-site intracellular action potential recording (Fig. 3c). The fabrication procedure commenced with the production of a multilayered two-dimensional precursor using standard micro/nanofabrication techniques, followed by its transfer and regional bonding onto a pre-strained elastomer substrate. Subsequently, the pre-strained elastomer substrate was released to generate a three-dimensional structure. The compression buckling technique enables the fabrication of transistor arrays with different layouts, sizes, and geometries across various scales. With different types of cardiac muscle cells (HL-1 cardiac muscle cells, neonatal mouse cell, adult mouse cell), the FET arrays successfully recorded intracellular action potentials with an amplitude of ~ 120 mV, which exhibited strong agreement in terms of both amplitude and morphology when compared to those detected by patch clamp. The novel fabrication process significantly enhances the performance of the FET, demonstrating the exceptional capability for precise coupling and faithful recording of intracellular action potential.

While silicon is used to fabricate three-dimensional nano-transistor, carbon-based three-dimensional nano-transistor also play an important role in identifying biochemical properties and intracellular measurements of individual living cells. Yuri Korche's team reported that the nano-sized dual-carbon electrodes can be fabricated by depositing pyrolytic carbon into quartz nanopipettes (Fig. 3d) [109]. Following the deposition of a thin layer of semiconductor material at the tip of the spear-shaped double carbon nanoelectrode, nanoscale field effect transistors with two individually addressable electrodes acting as drain and source can be produced. Immobilization of suitably recognized biomolecules on the semiconductor transistor channels produces selective field effect transistor biosensors. Three-dimensional active nano-transistors of different materials offer more possibilities for bioelectronic devices.

Three-dimensional bioelectronic devices can be fabricated from different materials and processes. Compared to two-dimensional bioelectronic devices, three-dimensional bioelectronics can improve biointerfacial coupling and increase the sealing resistance of cells-electrodes to reduce signal loss. Moreover, the three-dimensional electrodes can reduce the cell interface impedance and improve the signal acquisition efficiency. Twisted nanowire probes demonstrate the great potential of three-dimensional nano-transistor to interrogate biological systems by breaking through the barrier of designing on a flat surface. This three-dimensional nano-transistor exhibits conductivity and sensitivity in an aqueous solution, allowing stable intracellular recording. However, this twisted structure and device design limits the potential for probe size and multiplexing. The subsequently proposed branching intracellular nanotube FETs can overcome this limitation. These FETs possess a feature that allows the direct fabrication of multiple independent devices, thus enabling multiplexed recording of individual cells and cellular networks. In the future, further work is necessary to make three-dimensional nano-transistor as a routine tool, (i) designing small sizes and biomimetic coatings to minimize mechanical invasion of cells, (ii) improving signal-to-noise ratios to reach the level of the patch-clamp, and (iii) realizing higher density multiplexed recordings on high-density integrated planar nano-transistor, all of which will greatly expand the field of basic and applied electrophysiology research.

### Planar Active Micro-Transistor

**Fig. 4:**
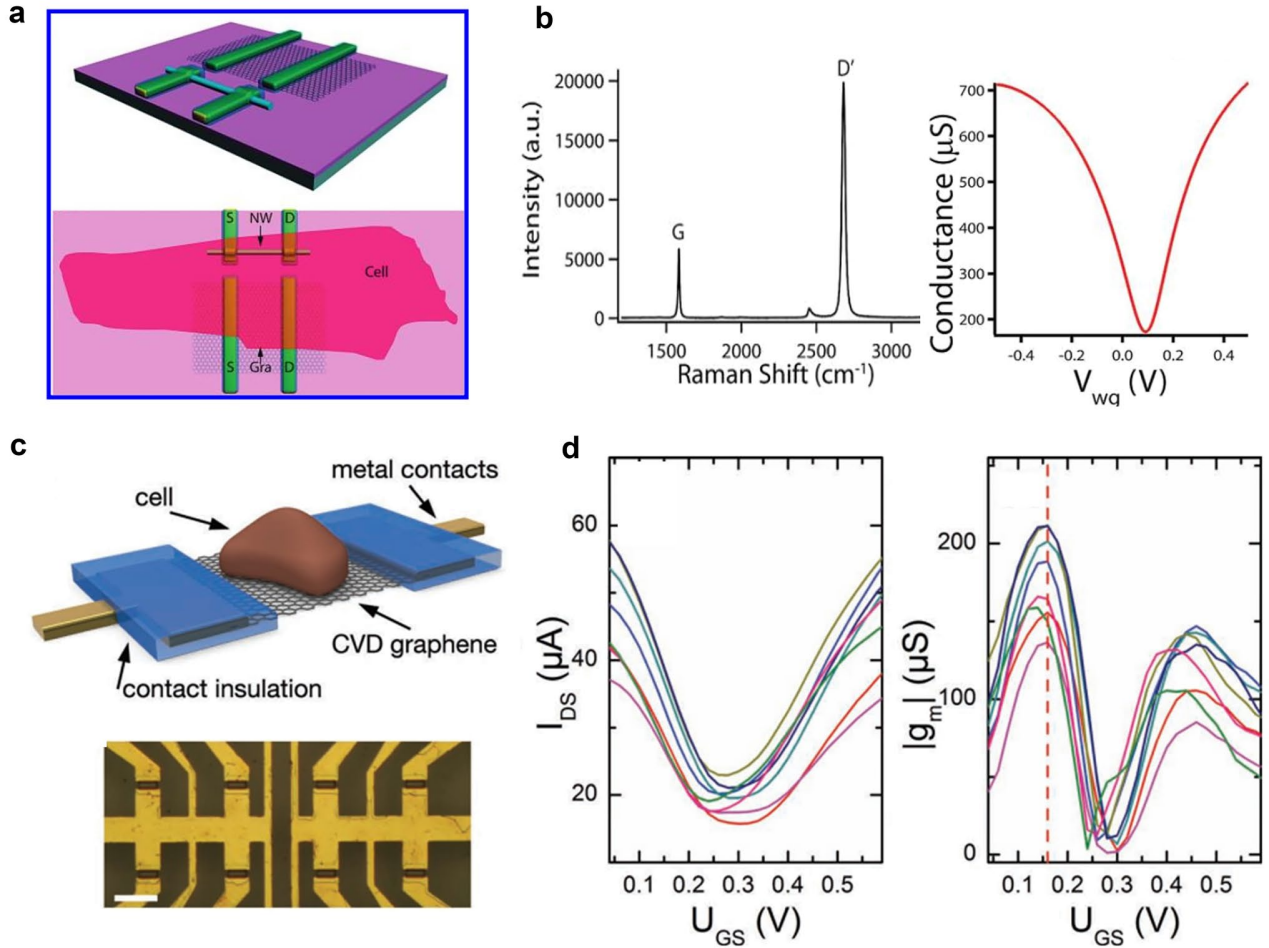
**a** Schematic diagram of graphene micro-transistor and Si-FET designs. **b** Raman spectra correspond to graphene micro-transistor (*left*) and the water-gate response of a typical graphene micro-transistor (*right*). Reproduced with permission from [40]. Copyright 2010, American Chemical Society. **c** Schematic diagram of the graphene micro-transistor, with graphene located between the drain and source metal contacts (*top*) and the microscope image of the eight transistors in the graphene micro-transistor array (*bottom*). Scale bar:100 μm. **d** Transistor current-gate voltage curves and corresponding transconductance-gate voltage curves of eight different devices in the same micro-transistor array. Reproduced with permission from [120]. Copyright 2011, WILEY–VCH

The preparation methodology for micro-transistor is distinct from that of nano-transistor. The Fiber group previously employed mechanical exfoliation to transfer monolayer graphene flakes onto a Si oxide substrate, followed by electron beam lithography (EBL) to identify the source/drain contacts, Cr/Au/Cr metallization, and SiO_2_ passivation of the contacts [110–113]. Fabricating the single atom thickness of the micro-transistor does exhibit better performance by simply changing the water gate potential (*V*_wg_) and does provide the unique ability to record signals from P-type and N-type devices. The present study reports the novel application of micro-transistor in recording clear electrophysiological signals from chicken embryonic cardiomyocytes, indicating the potential of micro-transistor in cellular electrophysiology (Fig. 4a, b) [40]. The observed variation in *V*_wg_ highlights the modulation of conductance signal amplitude by nearly an order of magnitude while maintaining a stable graphene/cell interface [91, 114–117].

In addition, Jose A. Garrido et al. used a graphene-based solution field effect transistor (GSFET) to detect cell signals (Fig. 4c, d). The arrays were fabricated using chemical vapor deposition (CVD) to grow large-area graphene films on copper foil, which provided a more advantageous technological platform for the preparation of graphene flakes due to their size and low-cost feasibility for large-scale production [118]. The sensor was found to support the growth of HL-1 cells effectively, highlighting the potential of this approach to detect cell signals. The propagation of cellular signals was successfully tracked using complete transistor arrays. The regulation of graphene's conductance in an electrolyte can be achieved by the application of gate voltage between the graphene film and the reference electrode within the electrolyte. This voltage, when applied, alters the Fermi energy level of the graphene layer, resulting in a corresponding change in the conductance of the device. Electrolyte-gated FET is frequently utilized to describe such devices. In graphene, for example, the type of charge carriers changes as the Fermi energy level intersects the Dirac point, thus allowing electron and hole conduction [119, 120].

Compared with the conventional planar electrodes, the fabrication process of active planar electrodes is much simpler, because source and drain electrodes of active planar electrodes can be simultaneously fabricated on the same plane by chemical vapor deposition, molecular beam epitaxy, and pulsed laser deposition. On the other hand, active planar electrodes possess better mechanical flexibility due to the use of mostly organic materials. Meanwhile, the stability is also better than that of the conventional planar electrodes because of its simple structure. Therefore, active planar electrodes are more suitable for the fabrication and application of flexible electronic devices due to their better mechanical flexibility and stability.

To obtain stable and predictable intracellular access while maintaining cell viability, micro-nano-devices are required to be able to penetrate the cell membrane and form a tight coupling with the cell membrane. Currently, the main penetration methods for realizing intracellular access are electroporation [121, 122] and optoporation [123–125]. The electroporation strategy in conjunction with 3D nano-electrodes can improve the efficiency of intracellular recording because the nano-electrodes can form a tighter coupling with the cell. The surrounding electric field can be focused on the electrode tip, generating low-voltage cell membrane nanopores at the cell-device interface. However, due to the transient nature of electroporation that makes intracellular recording decay, there is no significant improvement to the electrode-cell interface. Therefore, its inherent flaws and limitations need to be addressed to ensure intracellular recording. Another plasma optical technique provides accurate and independent recording of cells in different regions. Cells cultured on nanoelectrodes can open transient nanopores in the cell membrane when stimulated by a short pulse of laser light. This has no effect on the cell-electrode seal and does not interfere with its spontaneous electrical activity. In contrast to electroporation, optoporation is also capable of recording both intracellular and extracellular potentials without relying on 3D structures. Moreover, stable intracellular recordings can be achieved because there is no recording interruption in optoporation.

## Application of Active Micro-Nano-Transistor

Different types of active micro-nano-transistors have been developed to obtain electrophysiological signals. Based on their characteristics, the application of micro-nano-transistors in cardiomyocytes could lead to breakthroughs in cardiology and neuroscience. Cellular electrophysiological recordings can explore the activity of cells and their networks, and the mechanisms of ion channels can be explored for disease modeling and drug screening. Secondly, the utilization of highly sensitive electrophysiological signal recording techniques enables the comprehensive investigation of subthreshold potentials or membrane oscillations. Lastly, when micro-nano-transistors are combined with flexible devices, the acquisition of electrical signals from biological tissues can be achieved with stable fidelity and precision. Moreover, three-dimensional active nano-transistors and planar active micro-transistors have demonstrated distinct advantages for various application purposes (as illustrated in Table 1), exhibiting significant advancements in both cardiac and neuroscience research.

**Table 1:** Comparison of nano-transistors and micro-transistors

Type	Material	Process	Type of electrical signal	Amplitude	Throughput	Application	Refs.
Nano-transistors	Si, SiO_2_	VLS, EBL, ALD, photolithography	Intracellular electrical signal	60–120 mV	1–100	Electrophysiology, Drug response	[39, 41, 44, 96]
Micro-transistors	Graphene	Mechanical exfoliation, CVD	Extracellular electrical signal	< 10 mV	1–10	Implantable flexible devices	[40, 112, 135]

### Three-Dimensional Active Nano-Transistors in Cardiac and Neurophysiological Applications

**Fig. 5:**
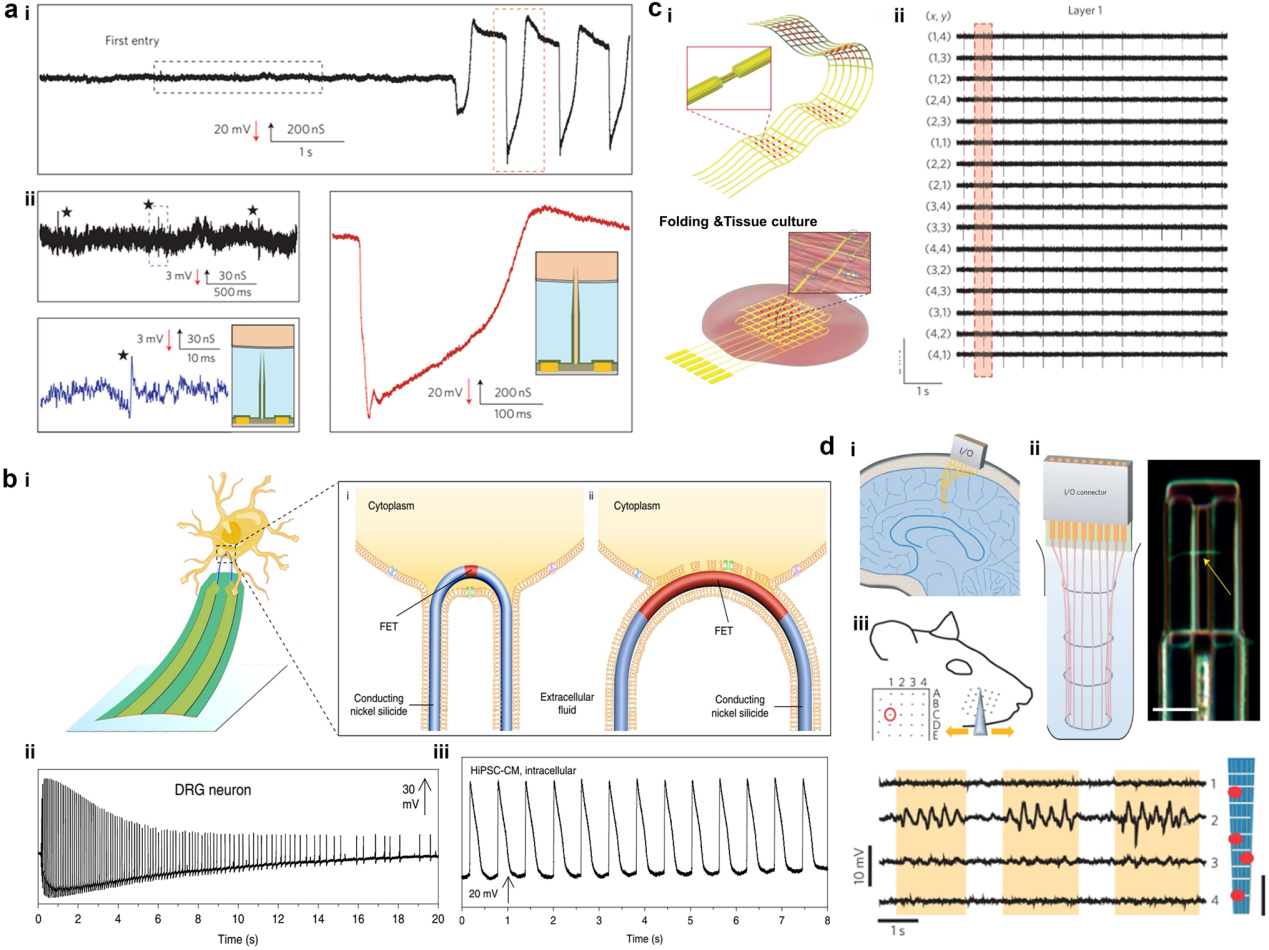
**a** (i) Typical transition from extracellular to intracellular recording (conductance versus time). (ii) The magnified view inside the black dashed box in (i) (*left*) and the magnified view in the red dashed box (*right*). (iii) Microscopic images of two BIT-FET devices coupled with cardiomyocytes and corresponding electrophysiological signal recordings. Reproduced with permission from [41]. Copyright 2012, Nature Publishing Group. **b** (i) Schematic representation of intracellular recording of U-NWFET. (ii) Intracellular signals recorded from a primary neuron as ROC curves of 0.75 μm for a FET probe with a channel length ∼ of 50 nm. (iii) The extracellular electrical signal of HiPSC-CMs recorded from a FET with ~ 2000 nm channel length and 1.5 μm ROC (*left*) and the intracellular electrical signal of hiPSC-CMs recorded from a FET with ~ 50 nm channel length and 0.75 μm ROC (*s*). Reproduced with permission from [105]. Copyright 2019, Nature Publishing Group. **c** (i) Schematic of a nanoelectronic scaffold with nanowire field effect transistor arrays and a 3D folded scaffold co-cultured with cardiac tissue. (ii) Electrical signal patterns recorded by 16 sensors in a nanoelectronic scaffold [73]. Copyright 2016, Nature Publishing Group. **d** (i) Schematic of implanted probe in the brain. (ii) Schematic of a large hole probe and a micrograph of a typical nanowire field effect transistor. Scale bar, 5 µm. (iii) Recorded mapping with a nanowire FET sensor in the cortical region of the rat brain. Scale bar, 200 µm [74]. Copyright 2015, Macmillan Publishing Group

Cardiomyocytes serve as the principal functional constituent of the heart, featuring a distinctive membrane structure comprising a phospholipid bilayer and membrane proteins that regulate ion transport, sustain intracellular homeostasis, and generate basal action potentials through specific ion channels [126, 127]. The transmembrane potential of cardiac myocytes can be categorized into resting and action potentials based on their respective properties. During the resting state, cell membrane is in polarization and its potential across the membrane is ~ −90 mV [128–130]. The autonomous cardiomyocytes generate the action potential spontaneously, which is subsequently transmitted to the working cardiomyocytes. The process of generating an action potential in cardiomyocytes can be segmented into five distinct phases, which include phase 0 (characterized by rapid depolarization due to inward flow of Na^+^), phase 1 (marked by early rapid repolarization due to outward flow of K^+^), phase 2 (a plateau phase resulting from the combined effects of outward flow of K^+^ and inward flow of Ca^2+^), phase 3 (late rapid repolarization due to outward flow of K^+^), and phase 4 (involving the restoration of intra- and extracellular concentrations of Na^+^, K^+^, and Ca^2+^ primarily through the action of ion pumps) [131–133]. The action potentials obtained by using patch-clamp measurements are typically ~ 100 mV and the duration of the action potential is ~ 200–400 ms [132]. It is noteworthy that the morphology of action potentials can be influenced by distinct cell types, including pacemakers, ventricular myocytes, and atrial myocytes, which exhibit variations in duration and under-phase. Furthermore, the high-sealing resistance and low interface impedance enhance the fidelity of the recorded signals. Presently, intracellular recordings utilizing three-dimensional nano-transistors represent the most accurate approximation of authentic action potentials. BIT-FETs, which are intracellular nanotube FETs, demonstrated exceptional action potential recordings from cardiac myocytes, exhibiting amplitudes of 75–100 mV and durations of ~ 200 ms (Fig. 5a) [41, 81]. The scalability and minimally invasive properties of nanodevices are crucial for the concurrent, prolonged monitoring of cardiac myocytes. The deterministic shape-controlled nanowire, coupled with spatially defined semiconductor-to-metal conversion, facilitated the production of scalable three-dimensional nano-transistors with controlled tip geometry and sensor size, thereby enabling the recording of intracellular action potentials of up to 100 mV from primary neurons **(**Fig. 5b(ii)) [42, 105]. Empirical investigations of neurons (Fig. 5b(ii)) and cardiomyocytes (Fig. 5b(iii)) have demonstrated that the regulation of device curvature and sensor dimensions is crucial for attaining intracellular recordings with elevated amplitude. The U-NWFET configuration permits multifaceted recordings from individual cells and cellular networks, thereby facilitating prospective inquiries into the dynamics of the brain and other tissues. Intracellular signals provide insights into cell types and the correlation between ion channel densities and cellular pathology in disease states. Subthreshold signals have the potential to uncover intercellular synchronization processes and electrophysiological regulatory mechanisms, which have significant implications for comprehending cellular physiology, pathology, and intercellular interactions [31, 134].

Moreover, three-dimensional active nano-transistors have also been extensively studied in three-dimensional tissue electrophysiology, which overcomes the critical drawbacks of the relatively low temporal resolution of optical voltage sensing and the planar electrode devices that are only studied on the surface of cells or tissue samples, demonstrating their potential in regenerative medicine and pharmacology. Lieber's group presented a three-dimensional nanoelectronic array of 64 sites with subcellular size and sub-millisecond temporal resolution. Real-time extracellular action potential recordings revealed quantitative patterning of action potential propagation in three-dimensional cardiac tissue [73]. The experiments also demonstrated that synchronized multisite stimulation and mapping can control the frequency and direction of action potential propagation, providing a new approach to cardiac electrophysiology with spatiotemporal recordings **(**Fig. 5c**)**. Likewise, three-dimensional active nano-transistors have the same potential for applications in neuroscience. A three-dimensional macroporous nanoelectronic brain probe with a high degree of porosity and cellular/subcellular feature sizes can optimize the neuron/probe interface and facilitate integration with the brain tissue, enabling the minimization of mechanical perturbations [74]. The results successfully recorded multiplexed local field potentials and single-cell action potentials in rat somatosensory cortex. As research on three-dimensional nano-transistor becomes more mature, the applications in cardiology and basic neuroscience are becoming more widespread, and the monitoring capabilities can be extended by integrating more properties in later studies **(**Fig. 5d**)**.

### Planar Active Micro-Transistors in Cardiac and Neural Networks Applications

The field of graphene micro-transistors is experiencing rapid growth and presents promising opportunities for the development of flexible and biocompatible devices that can interface with biological cells, particularly brain tissue [135–137]. When implemented in a secure operating environment, planar micro-transistors can be effectively integrated with bio-systems to enable real-time monitoring of both intra- and extracellular phenomena [84, 138, 139]. In addition, planar micro-transistors can be stabilized in direct contact with cells, allowing for accurate recording and amplification of electrical signals. The exceptional malleability of monolayer graphene enables its integration into pliable substrates, thereby presenting a novel technological approach for the development of implantable flexible devices possessing commendable bioactivity [140, 141]. The amalgamation of graphene with neural networks has been demonstrated to have no discernible impact on the fidelity of neuronal signals, nor does it impede the functionality of neural cells or tissues.

**Fig. 6:**
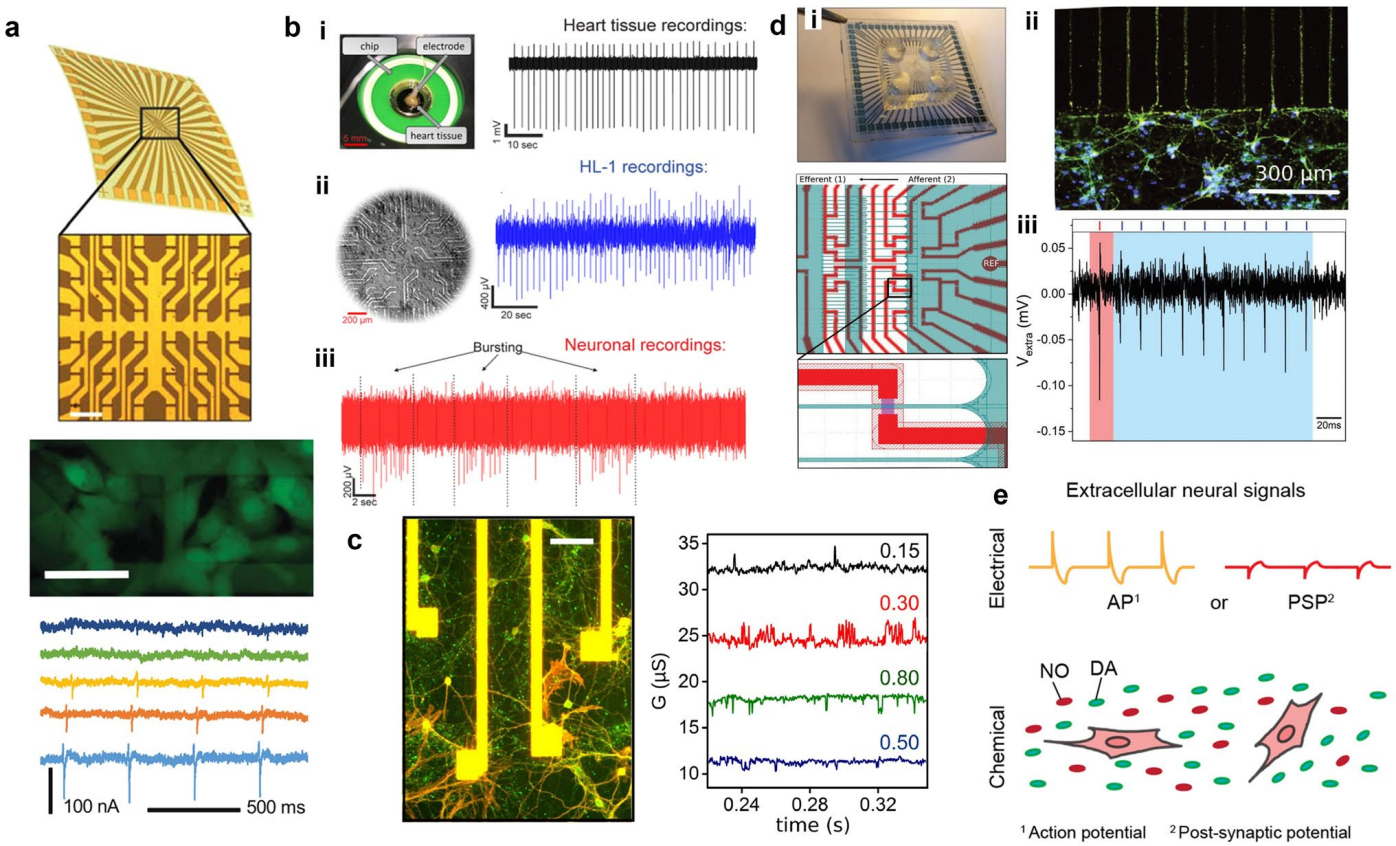
**a** Schematic of a flexible GFET on a polyimide substrate (*left*); Fluorescence images of HL-1 cells in flexible GFET and electrophysiological signals recorded. Scale bar: 60 μm. Reproduced with permission from [119]. Copyright 2016, Institute of Physics Publishing. **b** (i) Photographs of cardiac tissues and recorded electrophysiological signal traces recorded by GFET. (ii) Optical micrographs and typical electrophysiological signal traces of HL-1 cells cultured on GFET. (iii) Temporal recording trajectories of neurons. Reproduced with permission from [111]. Copyright 2017, Nature Publishing Group. **c** Optical micrographs of neurons on a 40 µm × 250 µm graphene transistor (scale size 40 µm) and conductance-time curves recorded from multiple gates [142]. Copyright 2018, Institute of Physics Publishing Group. **d** (i) Optical micrographs of GFET arrays combined with microfluidics and device layout for fluidic microchannels on a transistor. (ii) Immunofluorescence image of neurons cultured on graphene transistor. (iii) Electrical signal recording profiles of neurons cultured on graphene transistors [144]. Copyright 2022, Wiley–VCH Publishing Group. **e** During cell signaling, extracellular electrical signals are recorded electrically or chemically [147]. Copyright 2018, Institute of Physics Publishing Group

Planar micro-transistors have demonstrated their performance in measuring cardiac electrophysiological signals. In 2010, the liber group combined micro-transistors and silicon nano-transistors to detect electrophysiological signals from individual cardiomyocytes [40]. Micro-transistors can detect extracellular electrical signals with SNR typically exceeding 4, comparable to three-dimensional nano-transistors. The inter-peak width of narrow graphene micro-transistors (≈2 μm × 3 μm) is similar to that of nano-transistors (each area is approximately 100 times smaller). However, large-size graphene micro-transistors (≈20 μm × 10 μm) produce wider peak-to-peak signal widths. Then Jose A Garrido et al. in 2016 (Fig. 6a) employed flexible graphene micro-transistors to similarly measure the electrophysiological signals of cardiomyocytes, demonstrating that GSFET based on CVD graphene on polyimide substrates exhibit high conductance, low electronic noise, and no degradation [119]. As shown, the currents of five different FETs are demonstrated. The differences in cell signal propagation in the confluent cell layer and the coupling between the cell and each transistor make the recording time, action potential amplitude, and shape of the electrophysiological signals detected by the graphene micro-transistors different. The utilization of flexible graphene micro-transistors presents a promising avenue for the advancement of electroactive flexible implants in the future.

Furthermore, Andreas Offenhäusser et al. have proposed an electrochemical annealing/washing effect derived from in-plane external gate leakage currents that can record external potentials from isolated cardiac tissue and the cell line of HL-1 (Fig. 6b) [111]. The signals showed discernible action potentials with SNR greater than 14 for both isolated tissue and cardiomyocyte-like cell lines and greater than 6 for isolated cardiomyocyte-like cell lines. In addition, graphene transistors were recorded for the first time with distinguishable neuronal signals in vitro. Cécile Delacour et al. reported field-effect detection of ion channel activity in a network of neurons cultured for several weeks above an array of graphene transistors. The dependence of graphene transistor on potential gating is demonstrated as shown in Fig. 6c, where the amplitude of the signal (a few µS) remains roughly constant over a range of gate potentials from V_LG_ = 0 to 0.8 V. At the resolution of single-ion channel sensing, graphene transistor proves to be a promising platform for monitoring electrophysiological processes in living cells [43, 142].

Active micro-nano-transistors also have some limitations to restrict widespread use. Active bioelectronic devices require an external power source to control and amplify the current, and their fabrication and operation often require more-sophisticated techniques and equipment. In the future, active micro-nano-transistors can be combined with some emerging technologies, such as microfluidics, to expand their applications on electrophysiological and biochemical signals. Microfluidics is a promising technology for multimodal and prolonged recording of neuronal electrical activity by combining it with graphene field effect transistors, which is crucial for a better understanding of nervous system function and organization [143]. In their latest report, Cécile Delacour et al. utilized state-of-the-art graphene device arrays combined with microfluidics for culturing and sensing primary neurons [144]. The final structure of the neuronal network was assessed by immunofluorescence staining and calcium imaging, and the electrical maturity of the neurons was demonstrated by electrical signal recording (Fig. 6d). The combination of this novel platform offers the opportunity for versatile development of future diagnostics and therapeutics. In addition, various molecules, such as proteins, and gold nanorods [145, 146] can be selectively delivered to the target cells through microfluidic channels, while monitoring the electrical activity of cardiomyocytes/neurons and various biochemical signals, which provides a new approach for early pathology research (Fig. 6e) [147–149].

Optical methods have great potential in neuroscience to provide powerful tools for imaging and modulating physiological processes in the brain. Optical methods in the near-infrared (NIR) exhibit good deep-tissue penetration and low tissue absorption properties, providing a promising strategy for the recording of membrane potentials [150]. The optofluidic approach combined Raman spectroscopy with microfluidics is also a novel application to capture different species of microorganisms and classify them by artificial neural networks [151]. In addition, the utilization of flexible and stretchable organic electronic materials, coupled with low-impedance and low-modulus conductive polymer electrodes, presents a viable approach to mitigate invasiveness in surgical procedures and record action potentials with a high signal-to-noise ratio. This device's multiplexing capabilities enable accurate neural localization [152–154]. The emergence of various devices further expands the scope of electrophysiologic recording applications, providing robust technological resources for the diagnosis and treatment of cardiovascular and neurological disorders.

## Conclusion and Perspective

Advances in active micro/nano-bioelectronic devices are of great importance for enabling electrophysiological signal recording. First, three-dimensional active nano-transistor devices exhibit independence from interfacial impedance, thereby enabling the recording of intracellular potentials with full amplitude. The nanoscale size of three-dimensional nanodevices facilitates minimally invasive intracellular recording. Second, graphene micro-transistors have good chemical stability, biocompatibility, and extremely high carrier mobility, enabling electrophysiological signal recording in the heart and nerves. Based on the advantages of field-effect transistor nanoelectronics, active micro/nano-bioelectronic devices are accurate and sensitive for obtaining electrophysiological information from the heart and nerves and have been broadly applied to disease modeling and mapping of cellular networks.

Looking at the future, the development and enhancement of field-effect transistors for electrophysiological signal recording, particularly in neuroscience, presents numerous challenges and opportunities. In the diagnosis and treatment of cardiovascular and neurological diseases, micro-nano active transistors are required for specific targeting, multiplexing experiments, deep tissue/cellular insertion, and sub-cellular resolution detection. Electrophysiological signals reveal information about the cell type and density of various ion channels and are highly correlated with the disease state of the cell. Therefore, micro-nano-transistors can be used to obtain higher-quality electrophysiological signals by reducing device size and improving sensitivity, leading to a better understanding of cellular pathology and guiding therapies in many evolutionary disease models. In the case of nano-transistors, there exists a requirement to optimize device structure, enhance performance, and streamline fabrication processes. The intricate nature of active micro/nano-bioelectronic devices poses a significant obstacle to their mass production, and their high noise levels impede the accurate recording of weak and resting potentials without stimulation functionality. Sensitivity is important to reveal the vast amount of information contained in subthreshold activity to explore changes in neuronal loop behavior and action potential configuration. On the other hand, it is recommended that forthcoming research on biosensors for graphene micro-transistors should prioritize the distinctive physical and chemical characteristics provided by graphene, to enhance their sensitivity within the current need for dependability and consistency. For example, to attain multiplexed cell recording at the individual cell level, it is imperative to mitigate modular crosstalk between multiplexed graphene micro-transistor arrays and minimize device or environmental noise. In addition, potential advancements in the creation of flexible graphene micro-transistor cell sensors can be explored by integrating the exceptional electronic properties of graphene micro-transistor with the remarkable flexibility of graphene. With the ongoing advancements in technology, it is conceivable that the development of active bioelectronic devices will facilitate large-scale in vivo studies of neuronal/cardiac loop dynamics that could serve as a substitute for impaired neural tissue in the treatment of brain and paralytic diseases. This development would provide a breakthrough in research related to cardiology and neuroscience.

## References

[1] M. Jia, H. Dechiruji, J. Selberg, P. Pansodtee, J. Mathews et al., Bioelectronic control of chloride ions and concentration with Ag/AgCl contacts. APL Mater. **8**, 091106 (2020). 10.1063/5.0013867

[2] P.R.F. Rocha, P. Schlett, U. Kintzel, V. Mailänder, L.K.J. Vandamme et al., Electrochemical noise and impedance of Au electrode/electrolyte interfaces enabling extracellular detection of glioma cell populations. Sci. Rep. **6**, 34843 (2016). 10.1038/srep34843 27708378 10.1038/srep34843 PMC5052567

[3] J. Dunlop, M. Bowlby, R. Peri, D. Vasilyev, R. Arias, High-throughput electrophysiology: an emerging paradigm for ion-channel screening and physiology. Nat. Rev. Drug Discov. **7**, 358–368 (2008). 10.1038/nrd2552 18356919 10.1038/nrd2552

[4] R. Liu, R. Chen, A.T. Elthakeb, S.H. Lee, S. Hinckley et al., High density individually addressable nanowire arrays record intracellular activity from primary rodent and human stem cell derived neurons. Nano Lett. **17**, 2757–2764 (2017). 10.1021/acs.nanolett.6b04752 28384403 10.1021/acs.nanolett.6b04752 PMC6045931

[5] S. Sundelacruz, M. Levin, D.L. Kaplan, Role of membrane potential in the regulation of cell proliferation and differentiation. Stem Cell Rev. Rep. **5**, 231–246 (2009). 10.1007/s12015-009-9080-2 19562527 10.1007/s12015-009-9080-2 PMC10467564

[6] D.J. Blackiston, K.A. McLaughlin, M. Levin, Bioelectric controls of cell proliferation: ion channels, membrane voltage and the cell cycle. Cell Cycle **8**, 3527–3536 (2009). 10.4161/cc.8.21.9888 19823012 10.4161/cc.8.21.9888 PMC2862582

[7] A. Timmis, N. Townsend, C. Gale, R. Grobbee, N. Maniadakis et al., European society of cardiology: Cardiovascular disease statistics 2017. Oxford University Press, Oxford. (2018) 10.1093/eurheartj/ehx628 29190377

[8] Correction to: heart disease and stroke statistics-2023 update: a report from the American heart association. Circulation **148**, e4 (2023). 10.1161/CIR.0000000000001167 10.1161/CIR.0000000000001167 37486999

[9] G. Vorobiof, C. Silverstein, Non-invasive cardiac imaging for evaluation of cardiotoxicity in cancer patients-early detection and follow-up. SA Heart (2017). 10.24170/9-4-1829

[10] Y. Yang, A. Liu, C.-T. Tsai, C. Liu, J.C. Wu et al., Cardiotoxicity drug screening based on whole-panel intracellular recording. Biosens. Bioelectron. **216**, 114617 (2022). 10.1016/j.bios.2022.114617 36027802 10.1016/j.bios.2022.114617 PMC9930661

[11] L. Xiao, Z. Hu, W. Zhang, C. Wu, H. Yu et al., Evaluation of doxorubicin toxicity on cardiomyocytes using a dual functional extracellular biochip. Biosens. Bioelectron. **26**, 1493–1499 (2010). 10.1016/j.bios.2010.07.093 20732805 10.1016/j.bios.2010.07.093

[12] A.L. Hodgkin, A.F. Huxley, Action potentials recorded from inside a nerve fibre. Nature **144**, 710–711 (1939). 10.1038/144710a0

[13] L. Berdondini, K. Imfeld, A. Maccione, M. Tedesco, S. Neukom et al., Active pixel sensor array for high spatio-temporal resolution electrophysiological recordings from single cell to large scale neuronal networks. Lab Chip **9**, 2644–2651 (2009). 10.1039/B907394A 19704979 10.1039/b907394a

[14] C.-X. Lin, J.-L. Gu, J.-M. Cao, The acute toxic effects of platinum nanoparticles on ion channels, transmembrane potentials of cardiomyocytes *in vitro* and heart rhythm *in vivo* in mice. Int. J. Nanomedicine **14**, 5595–5609 (2019). 10.2147/IJN.S209135 31413565 10.2147/IJN.S209135 PMC6660630

[15] T. Meyer, K.-H. Boven, E. Günther, M. Fejtl, Micro-electrode arrays in cardiac safety pharmacology: a novel tool to study QT interval prolongation. Drug Saf. **27**, 763–772 (2004). 10.2165/00002018-200427110-00002 15350150 10.2165/00002018-200427110-00002

[16] D. Xu, J. Mo, X. Xie, N. Hu, In-cell nanoelectronics: opening the door to intracellular electrophysiology. Nano-Micro Lett. **13**, 127 (2021). 10.1007/s40820-021-00655-x 10.1007/s40820-021-00655-x PMC8124030 34138366

[17] J. Fang, S. Huang, F. Liu, G. He, X. Li et al., Semi-implantable bioelectronics. Nano-Micro Lett. **14**, 125 (2022). 10.1007/s40820-022-00818-4 10.1007/s40820-022-00818-4 PMC9148344 35633391

[18] D. Ossola, M.-Y. Amarouch, P. Behr, J. Vörös, H. Abriel et al., Force-controlled patch clamp of beating cardiac cells. Nano Lett. **15**, 1743–1750 (2015). 10.1021/nl504438z 25639960 10.1021/nl504438z

[19] B. Hille, Ion channels of excitable membranes sunderland. Sinauer Associates Inc. (2001)

[20] A. Molleman, Patch clamping: an introductory guide to patch clamp electrophysiology (Patch Clamping: An Introductory Guide To Patch Clamp Electrophysiology; 2003)

[21] D. C. Sigg, P. A. Iaizzo, Y. F. Xiao, B. He. Electrophysiology of single cardiomyocytes: Patch clamp and other recording methods. (Chapter 16), 329–348 (2010). 10.1007/978-1-4419-6658-2_16

[22] R.L. Schrøder, M. Christensen, B. Anson, M. Sunesen, Exploring stem cell-derived cardiomyocytes with automated patch clamp techniques. Biophys. J. **102**, 544a (2012). 10.1016/j.bpj.2011.11.2968

[23] B. Amuzescu, S. Frech, K. Lin, J. Eisfeld, J. Kudolo et al., Electrophysiology Characterization of Human Induced Pluripotent Stem Cell-derived Cardiomyocytes Using Automated Patch-clamp. (2015) 10.1089/adt.2014.601 25353059

[24] A. Marques-Smith, J.P. Neto, G. Lopes, J. Nogueira, L. Calcaterra et al., Recording from the same neuron with high-density CMOS probes and patch-clamp: a ground-truth dataset and an experiment in collaboration. bioRxiv (2018). 10.1101/370080

[25] V. Grenier, K.N. Martinez, B.R. Benlian, D.M. García-Almedina, B.K. Raliski et al., Molecular prosthetics for long-term functional imaging with fluorescent reporters. ACS Cent. Sci. **8**, 118–121 (2022). 10.1021/acscentsci.1c01153 35111902 10.1021/acscentsci.1c01153 PMC8802189

[26] A. Grinvald, R. Hildesheim, VSDI: a new era in functional imaging of cortical dynamics. Nat. Rev. Neurosci. **5**, 874–885 (2004). 10.1038/nrn1536 15496865 10.1038/nrn1536

[27] L.N. Kahyaoglu, R. Madangopal, M. Stensberg, Rickus J.L, Light-directed functionalization methods for high-resolution optical fiber based biosensors. SPIE Sensing Technology + Applications. Proc SPIE 9486, Advanced Environmental, Chemical, and Biological Sensing Technologies XII Baltimore, MD, USA **9486**, 9–18 (2015). 10.1117/12.2177178

[28] A. Matiukas, B.G. Mitrea, M. Qin, A.M. Pertsov, A.G. Shvedko et al., Near-infrared voltage-sensitive fluorescent dyes optimized for optical mapping in blood-perfused myocardium. Heart Rhythm **4**, 1441–1451 (2007). 10.1016/j.hrthm.2007.07.012 17954405 10.1016/j.hrthm.2007.07.012 PMC2121222

[29] M. Warren, K.W. Spitzer, B.W. Steadman, T.D. Rees, P. Venable et al., High-precision recording of the action potential in isolated cardiomyocytes using the near-infrared fluorescent dye di-4-ANBDQBS. Am. J. Physiol. Heart Circ. Physiol. **299**, H1271–H1281 (2010). 10.1152/ajpheart.00248.2010 20601458 10.1152/ajpheart.00248.2010 PMC2957348

[30] M. Warren, K.W. Spitzer, B.W. Steadman, P. Venable, T. Taylor et al., Near infrared emitting dye di-4-ANBDQBS for recording action potentials in isolated cardiomyocytes. Biophys. J. **96**, 293a (2009). 10.1016/j.bpj.2008.12.1453

[31] J. Abbott, T. Ye, K. Krenek, R.S. Gertner, S. Ban et al., A nanoelectrode array for obtaining intracellular recordings from thousands of connected neurons. Nat. Biomed. Eng. **4**, 232–241 (2020). 10.1038/s41551-019-0455-7 31548592 10.1038/s41551-019-0455-7 PMC7035150

[32] T. Banno, S. Tsuruhara, Y. Seikoba, R. Tonai, K. Yamashita et al., Nanoneedle-electrode devices for *in vivo* recording of extracellular action potentials. ACS Nano **16**, 10692–10700 (2022). 10.1021/acsnano.2c02399 35786946 10.1021/acsnano.2c02399

[33] A. Barbaglia, M. Dipalo, G. Melle, G. Iachetta, L. Deleye et al., Mirroring action potentials: label-free, accurate, and noninvasive electrophysiological recordings of human-derived cardiomyocytes. Adv. Mater. **33**, e2004234 (2021). 10.1002/adma.202004234 33410191 10.1002/adma.202004234 PMC11468743

[34] B.X.E. Desbiolles, E. de Coulon, A. Bertsch, S. Rohr, P. Renaud, Intracellular recording of cardiomyocyte action potentials with nanopatterned volcano-shaped microelectrode arrays. Nano Lett. **19**, 6173–6181 (2019). 10.1021/acs.nanolett.9b02209 31424942 10.1021/acs.nanolett.9b02209

[35] J. Fang, D. Xu, H. Wang, J. Wu, Y. Li et al., Scalable and robust hollow nanopillar electrode for enhanced intracellular action potential recording. Nano Lett. **23**, 243–251 (2023). 10.1021/acs.nanolett.2c04222 36537828 10.1021/acs.nanolett.2c04222

[36] Z. Jahed, Y. Yang, C.-T. Tsai, E.P. Foster, A.F. McGuire et al., Nanocrown electrodes for parallel and robust intracellular recording of cardiomyocytes. Nat. Commun. **13**, 2253 (2022). 10.1038/s41467-022-29726-2 35474069 10.1038/s41467-022-29726-2 PMC9042818

[37] J.T. Robinson, M. Jorgolli, A.K. Shalek, M.-H. Yoon, R.S. Gertner et al., Vertical nanowire electrode arrays as a scalable platform for intracellular interfacing to neuronal circuits. Nat. Nanotechnol. **7**, 180–184 (2012). 10.1038/nnano.2011.249 22231664 10.1038/nnano.2011.249 PMC4209482

[38] M. Abarkan, A. Pirog, D. Mafilaza, G. Pathak, G. N’Kaoua et al., Vertical organic electrochemical transistors and electronics for low amplitude micro-organ signals. Adv. Sci. **9**, e2105211 (2022). 10.1002/advs.202105211 10.1002/advs.202105211 PMC8922095 35064774

[39] B. Tian, T. Cohen-Karni, Q. Qing, X. Duan, P. Xie et al., Three-dimensional, flexible nanoscale field-effect transistors as localized bioprobes. Science **329**, 830–834 (2010). 10.1126/science.1192033 20705858 10.1126/science.1192033 PMC3149824

[40] T. Cohen-Karni, Q. Qing, Q. Li, Y. Fang, C.M. Lieber, Graphene and nanowire transistors for cellular interfaces and electrical recording. Nano Lett. **10**, 1098–1102 (2010). 10.1021/nl1002608 20136098 10.1021/nl1002608 PMC2899684

[41] X. Duan, R. Gao, P. Xie, T. Cohen-Karni, Q. Qing et al., Intracellular recordings of action potentials by an extracellular nanoscale field-effect transistor. Nat. Nanotechnol. **7**, 174–179 (2011). 10.1038/nnano.2011.223 22179566 10.1038/nnano.2011.223 PMC3293943

[42] Q. Qing, Z. Jiang, L. Xu, R. Gao, L. Mai et al., Free-standing kinked nanowire transistor probes for targeted intracellular recording in three dimensions. Nat. Nanotechnol. **9**, 142–147 (2014). 10.1038/nnano.2013.273 24336402 10.1038/nnano.2013.273 PMC3946362

[43] S. Asgarifar, H. Gomes, A. Mestre, P.M. C. Inácio, J. Bragança et al., in Electrochemically Gated Graphene Field-effect Transistor for Extracellular Cell Signal Recording. ed. by (2016), pp. 558–564.

[44] Y. Gu, C. Wang, N. Kim, J. Zhang, T.M. Wang et al., Three-dimensional transistor arrays for intra- and inter-cellular recording. Nat. Nanotechnol. **17**, 292–300 (2022). 10.1038/s41565-021-01040-w 34949774 10.1038/s41565-021-01040-w PMC8994210

[45] A. Kyndiah, F. Leonardi, C. Tarantino, T. Cramer, R. Millan-Solsona et al., Bioelectronic recordings of cardiomyocytes with accumulation mode electrolyte gated organic field effect transistors. Biosens. Bioelectron. **150**, 111844 (2020). 10.1016/j.bios.2019.111844 31740253 10.1016/j.bios.2019.111844

[46] H. Gao, F. Yang, K. Sattari, X. Du, T. Fu et al., Bioinspired two-in-one nanotransistor sensor for the simultaneous measurements of electrical and mechanical cellular responses. Sci. Adv. **8**, eabn2485 (2022). 10.1126/sciadv.abn2485 10.1126/sciadv.abn2485 PMC9401615 36001656

[47] P. Connolly, P. Clark, A.S.G. Curtis, J.A.T. Dow, C.D.W. Wilkinson, An Extracellular microelectrode Array for monitoring electrogenic cells in culture. Biosens. Bioelectron. **5**, 223–234 (1990). 10.1016/0956-5663(90)80011-2 2206490 10.1016/0956-5663(90)80011-2

[48] T.J. Blanche, M.A. Spacek, J.F. Hetke, N.V. Swindale, Polytrodes: high-density silicon electrode arrays for large-scale multiunit recording. J. Neurophysiol. **93**, 2987–3000 (2005). 10.1152/jn.01023.2004 15548620 10.1152/jn.01023.2004

[49] P.J. Koester, C. Tautorat, H. Beikirch, J. Gimsa, W. Baumann, Recording electric potentials from single adherent cells with 3D microelectrode arrays after local electroporation. Biosens. Bioelectron. **26**, 1731–1735 (2010). 10.1016/j.bios.2010.08.003 20800467 10.1016/j.bios.2010.08.003

[50] M.E. Spira, A. Hai, Multi-electrode array technologies for neuroscience and cardiology. Nat. Nanotechnol. **8**, 83–94 (2013). 10.1038/nnano.2012.265 23380931 10.1038/nnano.2012.265

[51] V. Zlochiver, S.L. Kroboth, C.R. Beal, J.A. Cook, R. Joshi-Mukherjee, Human iPSC-derived cardiomyocyte networks on multiwell micro-electrode arrays for recurrent action potential recordings. J. Vis. Exp. **149**, e59906 (2019). 10.3791/59906 10.3791/59906 31355788

[52] X. Wei, C. Qin, C. Gu, C. He, Q. Yuan et al., A novel bionic *in vitro* bioelectronic tongue based on cardiomyocytes and microelectrode array for bitter and umami detection. Biosens. Bioelectron. **145**, 111673 (2019). 10.1016/j.bios.2019.111673 31546200 10.1016/j.bios.2019.111673

[53] A. Zhang, C.M. Lieber, Nano-bioelectronics. Chem. Rev. **116**, 215–257 (2016). 10.1021/acs.chemrev.5b00608 26691648 10.1021/acs.chemrev.5b00608 PMC4867216

[54] I. Zadorozhnyi, H. Hlukhova, Y. Kutovyi, V. Handziuk, N. Naumova et al., Towards pharmacological treatment screening of cardiomyocyte cells using Si nanowire FETs. Biosens. Bioelectron. **137**, 229–235 (2019). 10.1016/j.bios.2019.04.038 31121460 10.1016/j.bios.2019.04.038

[55] G. Presnova, D. Presnov, V. Krupenin, V. Grigorenko, A. Trifonov et al., Biosensor based on a silicon nanowire field-effect transistor functionalized by gold nanoparticles for the highly sensitive determination of prostate specific antigen. Biosens. Bioelectron. **88**, 283–289 (2017). 10.1016/j.bios.2016.08.054 27567265 10.1016/j.bios.2016.08.054

[56] J. Abbott, T. Ye, L. Qin, M. Jorgolli, R.S. Gertner et al., CMOS nanoelectrode array for all-electrical intracellular electrophysiological imaging. Nat. Nanotechnol. **12**, 460–466 (2017). 10.1038/nnano.2017.3 28192391 10.1038/nnano.2017.3

[57] J.S. Park, S.I. Grijalva, D. Jung, S. Li, G.V. Junek et al., Intracellular cardiomyocytes potential recording by planar electrode array and fibroblasts co-culturing on multi-modal CMOS chip. Biosens. Bioelectron. **144**, 111626 (2019). 10.1016/j.bios.2019.111626 31494510 10.1016/j.bios.2019.111626 PMC8559116

[58] J. Müller, M. Ballini, P. Livi, Y. Chen, M. Radivojevic et al., High-resolution CMOS MEA platform to study neurons at subcellular, cellular, and network levels. Lab Chip **15**, 2767–2780 (2015). 10.1039/C5LC00133A 25973786 10.1039/c5lc00133a PMC5421573

[59] C.M. Lieber, Semiconductor nanowires: a platform for nanoscience and nanotechnology. MRS Bull. **36**, 1052–1063 (2011). 10.1557/mrs.2011.269 22707850 10.1557/mrs.2011.269 PMC3375735

[60] B.P. Timko, T. Cohen-Karni, Q. Qing, B. Tian, C.M. Lieber, Design and implementation of functional nanoelectronic interfaces with biomolecules, cells, and tissue using nanowire device arrays. IEEE Trans. Nanotechnol. **9**, 269–280 (2010). 10.1109/TNANO.2009.2031807 21785576 10.1109/TNANO.2009.2031807 PMC3140208

[61] P.B. Kruskal, Z. Jiang, T. Gao, C.M. Lieber, Beyond the patch clamp: nanotechnologies for intracellular recording. Neuron **86**, 21–24 (2015). 10.1016/j.neuron.2015.01.004 25856481 10.1016/j.neuron.2015.01.004

[62] Y. Zhang, L.F. Duan, Y. Zhang, J. Wang, H. Geng et al., Advances in conceptual electronic nanodevices based on 0D and 1D nanomaterials. Nano-Micro Lett. **6**, 1–19 (2014). 10.1007/BF03353763

[63] M. Liu, Z. Wu, W.M. Lau, J. Yang, Recent advances in directed assembly of nanowires or nanotubes. Nano-Micro Lett. **4**, 142–153 (2012). 10.1007/BF03353705

[64] Y. Fang, Y. Jiang, H. Acaron Ledesma, J. Yi, X. Gao et al., Texturing silicon nanowires for highly localized optical modulation of cellular dynamics. Nano Lett. **18**, 4487–4492 (2018). 10.1021/acs.nanolett.8b01626 10.1021/acs.nanolett.8b01626 PMC6814189 29894630

[65] P. Singh, S.K. Pandey, J. Singh, S. Srivastava, S. Sachan et al., Biomedical perspective of electrochemical nanobiosensor. Nano-Micro Lett. **8**, 193–203 (2016). 10.1007/s40820-015-0077-x 10.1007/s40820-015-0077-x PMC6223677 30460280

[66] J. Li, Y. Ma, D. Huang, Z. Wang, Z. Zhang et al., High-performance flexible microneedle array as a low-impedance surface biopotential dry electrode for wearable electrophysiological recording and polysomnography. Nano-Micro Lett. **14**, 132 (2022). 10.1007/s40820-022-00870-0 10.1007/s40820-022-00870-0 PMC9198145 35699782

[67] Y. Qiao, J. Luo, T. Cui, H. Liu, H. Tang et al., Soft electronics for health monitoring assisted by machine learning. Nano-Micro Lett. **15**, 66 (2023). 10.1007/s40820-023-01029-1 10.1007/s40820-023-01029-1 PMC10014415 36918452

[68] D. Jäckel, D.J. Bakkum, T.L. Russell, J. Müller, M. Radivojevic et al., Combination of high-density microelectrode array and patch clamp recordings to enable studies of multisynaptic integration. Sci. Rep. **7**, 978 (2017). 10.1038/s41598-017-00981-4 28428560 10.1038/s41598-017-00981-4 PMC5430511

[69] Y. Zhang, Y. Tang, Y. Wang, L. Zhang, Nanomaterials for cardiac tissue engineering application. Nano-Micro Lett. **3**, 270–277 (2011). 10.1007/BF03353683

[70] J. Lou-Franco, B. Das, C. Elliott, C. Cao, Gold nanozymes: from concept to biomedical applications. Nano-Micro Lett. **13**, 10 (2020). 10.1007/s40820-020-00532-z 10.1007/s40820-020-00532-z PMC8187695 34138170

[71] Y. Jin, H. Wang, K. Yi, S. Lv, H. Hu et al., Applications of nanobiomaterials in the therapy and imaging of acute liver failure. Nano-Micro Lett. **13**, 25 (2020). 10.1007/s40820-020-00550-x 10.1007/s40820-020-00550-x PMC8187515 34138224

[72] S. Kim, J. Seo, J. Choi, H. Yoo, Vertically integrated electronics: new opportunities from emerging materials and devices. Nano-Micro Lett. **14**, 201 (2022). 10.1007/s40820-022-00942-1 10.1007/s40820-022-00942-1 PMC9547046 36205848

[73] X. Dai, W. Zhou, T. Gao, J. Liu, C.M. Lieber, Three-dimensional mapping and regulation of action potential propagation in nanoelectronics-innervated tissues. Nat. Nanotechnol. **11**, 776–782 (2016). 10.1038/nnano.2016.96 27347837 10.1038/nnano.2016.96 PMC5014560

[74] C. Xie, J. Liu, T.-M. Fu, X. Dai, W. Zhou et al., Three-dimensional macroporous nanoelectronic networks as minimally invasive brain probes. Nat. Mater. **14**, 1286–1292 (2015). 10.1038/nmat4427 26436341 10.1038/nmat4427

[75] S.K. Krishnan, N. Nataraj, M. Meyyappan, U. Pal, Graphene-based field-effect transistors in biosensing and neural interfacing applications: recent advances and prospects. Anal. Chem. **95**, 2590–2622 (2023). 10.1021/acs.analchem.2c03399 36693046 10.1021/acs.analchem.2c03399 PMC11386440

[76] S. Wang, M.Z. Hossain, K. Shinozuka, N. Shimizu, S. Kitada et al., Graphene field-effect transistor biosensor for detection of biotin with ultrahigh sensitivity and specificity. Biosens. Bioelectron. **165**, 112363 (2020). 10.1016/j.bios.2020.112363 32729495 10.1016/j.bios.2020.112363 PMC7272179

[77] L. Xu, Z. Jiang, L. Mai, Q. Qing, Multiplexed free-standing nanowire transistor bioprobe for intracellular recording: a general fabrication strategy. Nano Lett. **14**, 3602–3607 (2014). 10.1021/nl5012855 24836976 10.1021/nl5012855

[78] C. Yao, Q. Li, J. Guo, F. Yan, I.-M. Hsing, Rigid and flexible organic electrochemical transistor arrays for monitoring action potentials from electrogenic cells. Adv. Healthc. Mater. **4**, 528–533 (2015). 10.1002/adhm.201400406 25358525 10.1002/adhm.201400406

[79] S.J. Luck, An introduction to the event-related potential technique. Sveučilište u Rijeci. (2005)

[80] S. Cabrini, Sub-10-nm three-dimensional plasmonic probes and sensors. 2016 Progress in Electromagnetic Research Symposium (PIERS). Shanghai, China. IEEE, (2016). p 836

[81] R. Gao, S. Strehle, B. Tian, T. Cohen-Karni, P. Xie et al., Outside looking in: nanotube transistor intracellular sensors. Nano Lett. **12**, 3329–3333 (2012). 10.1021/nl301623p 10.1021/nl301623p PMC3374901 22583370

[82] T.P. Dasari Shareena, D. McShan, A.K. Dasmahapatra, P.B. Tchounwou, A review on graphene-based nanomaterials in biomedical applications and risks in environment and health. Nano-Micro Lett. **10**, 53 (2018). 10.1007/s40820-018-0206-4 10.1007/s40820-018-0206-4 PMC6075845 30079344

[83] S. Luo, L. Peng, Y. Xie, X. Cao, X. Wang et al., Flexible large-area graphene films of 50–600nm thickness with high carrier mobility. Nano-Micro Lett. **15**, 61 (2023). 10.1007/s40820-023-01032-6 10.1007/s40820-023-01032-6 PMC9984600 36867262

[84] L. Tang, Y. Wang, Y. Li, H. Feng, J. Lu et al., Preparation, structure, and electrochemical properties of reduced graphene sheet films. Adv. Funct. Mater. **19**, 2782–2789 (2009). 10.1002/adfm.200900377

[85] W.C. Lee, C.H. Lim, H. Shi, L.A. Tang, Y. Wang et al., Origin of enhanced stem cell growth and differentiation on graphene and graphene oxide. ACS Nano **5**, 7334–7341 (2011). 10.1021/nn202190c 21793541 10.1021/nn202190c

[86] M. Kaisti, Detection principles of biological and chemical FET sensors. Biosens. Bioelectron. **98**, 437–448 (2017). 10.1016/j.bios.2017.07.010 28711826 10.1016/j.bios.2017.07.010

[87] W. Fu, L. Jiang, E.P. van Geest, L.M. Lima, G.F. Schneider, Sensing at the surface of graphene field-effect transistors. Adv. Mater. **29**, 1603610 (2017). 10.1002/adma.201603610 10.1002/adma.201603610 27896865

[88] R. Stine, S.P. Mulvaney, J.T. Robinson, C.R. Tamanaha, P.E. Sheehan, Fabrication, optimization, and use of graphene field effect sensors. Anal. Chem. **85**, 509–521 (2013). 10.1021/ac303190w 23234380 10.1021/ac303190w

[89] T. Feuk, On the transparency of the stroma in the mammalian *Cornea*. IEEE Trans. Biomed. Eng. **BME-17**, 186–190 (1970). 10.1109/tbme.1970.4502732 10.1109/tbme.1970.4502732 5464476

[90] S.-A. Peng, Z. Jin, P. Ma, D.-Y. Zhang, J.-Y. Shi et al., The sheet resistance of graphene under contact and its effect on the derived specific contact resistivity. Carbon **82**, 500–505 (2015). 10.1016/j.carbon.2014.11.001

[91] W. Fu, C. Nef, A. Tarasov, M. Wipf, R. Stoop et al., High mobility graphene ion-sensitive field-effect transistors by noncovalent functionalization. Nanoscale **5**, 12104–12110 (2013). 10.1039/C3NR03940D 24142362 10.1039/c3nr03940d

[92] L.H. Hess, M. Seifert, J.A. Garrido, Graphene transistors for bioelectronics. Proc. IEEE **101**, 1780–1792 (2013). 10.1109/JPROC.2013.2261031

[93] F. Veliev, Z. Han, D. Kalita, A. Briançon-Marjollet, V. Bouchiat et al., Recording spikes activity in cultured hippocampal neurons using flexible or transparent graphene transistors. Front. Neurosci. **11**, 466 (2017). 10.3389/fnins.2017.00466 28894412 10.3389/fnins.2017.00466 PMC5581354

[94] L. Xu, Z. Jiang, Q. Qing, L. Mai, Q. Zhang et al., Design and synthesis of diverse functional kinked nanowire structures for nanoelectronic bioprobes. Nano Lett. **13**, 746–751 (2013). 10.1021/nl304435z 23273386 10.1021/nl304435z PMC3572243

[95] Z. Jiang, Q. Qing, P. Xie, R. Gao, C.M. Lieber, Kinked p-n junction nanowire probes for high spatial resolution sensing and intracellular recording. Nano Lett. **12**, 1711–1716 (2012). 10.1021/nl300256r 22309132 10.1021/nl300256r PMC3303933

[96] T.-M. Fu, X. Duan, Z. Jiang, X. Dai, P. Xie et al., Sub-10-nm intracellular bioelectronic probes from nanowire–nanotube heterostructures. Proc. Natl. Acad. Sci. U.S.A. **111**, 1259–1264 (2014). 10.1073/pnas.1323389111 24474745 10.1073/pnas.1323389111 PMC3910633

[97] T. Cohen-Karni, D. Casanova, J.F. Cahoon, Q. Qing, D.C. Bell et al., Synthetically encoded ultrashort-channel nanowire transistors for fast, pointlike cellular signal detection. Nano Lett. **12**, 2639–2644 (2012). 10.1021/nl3011337 22468846 10.1021/nl3011337 PMC3348975

[98] R. Elnathan, M. Kwiat, F. Patolsky, N.H. Voelcker, Engineering vertically aligned semiconductor nanowire arrays for applications in the life sciences. Nano Today **9**, 172–196 (2014). 10.1016/j.nantod.2014.04.001

[99] J. Westwater, D.P. Gosain, S. Tomiya, S. Usui, H. Ruda, Growth of silicon nanowires via gold/silane vapor–liquid–solid reaction. J. Vac. Sci. Technol. B Microelectron. Nanometer Struct. Process. Meas. Phenom. **15**, 554–557 (1997). 10.1116/1.589291

[100] Q. Gao, V.G. Dubrovskii, P. Caroff, J. Wong-Leung, L. Li et al., Simultaneous selective-area and vapor-liquid-solid growth of InP nanowire arrays. Nano Lett. **16**, 4361–4367 (2016). 10.1021/acs.nanolett.6b01461 27253040 10.1021/acs.nanolett.6b01461

[101] S. Barth, F. Hernandez-Ramirez, J.D. Holmes, A. Romano-Rodriguez, Synthesis and applications of one-dimensional semiconductors. Prog. Mater. Sci. **55**, 563–627 (2010). 10.1016/j.pmatsci.2010.02.001

[102] J. Hu, T.W. Odom, C.M. Lieber, Chemistry and physics in one dimension: synthesis and properties of nanowires and nanotubes. Acc. Chem. Res. **32**, 435–445 (1999). 10.1021/ar9700365

[103] A.K. Shalek, J.T. Robinson, E.S. Karp, J.S. Lee, D.-R. Ahn et al., Vertical silicon nanowires as a universal platform for delivering biomolecules into living cells. Proc. Natl. Acad. Sci. U.S.A. **107**, 1870–1875 (2010). 10.1073/pnas.0909350107 20080678 10.1073/pnas.0909350107 PMC2836617

[104] Y.J. Hwang, C. Hahn, B. Liu, P. Yang, Photoelectrochemical properties of TiO_2_ nanowire arrays: a study of the dependence on length and atomic layer deposition coating. ACS Nano **6**, 5060–5069 (2012). 10.1021/nn300679d 22621345 10.1021/nn300679d

[105] Y. Zhao, S.S. You, A. Zhang, J.H. Lee, J. Huang et al., Scalable ultrasmall three-dimensional nanowire transistor probes for intracellular recording. Nat. Nanotechnol. **14**, 783–790 (2019). 10.1038/s41565-019-0478-y 31263191 10.1038/s41565-019-0478-y

[106] Z. Huang, H. Fang, J. Zhu, Fabrication of silicon nanowire arrays with controlled diameter, length, and density. Adv. Mater. **19**, 744–748 (2007). 10.1002/adma.200600892

[107] Y.Q. Fu, A. Colli, A. Fasoli, J.K. Luo, A.J. Flewitt et al., Deep reactive ion etching as a tool for nanostructure fabrication. J. Vac. Sci. Technol. B Microelectron. Nanometer Struct. Process. Meas. Phenom. **27**, 1520–1526 (2009). 10.1116/1.3065991

[108] R. Juhasz, N. Elfström, J. Linnros, Controlled fabrication of silicon nanowires by electron beam lithography and electrochemical size reduction. Nano Lett. **5**, 275–280 (2005). 10.1021/nl0481573 15794610 10.1021/nl0481573

[109] Y. Zhang, J. Clausmeyer, B. Babakinejad, A.L. Córdoba, T. Ali et al., Spearhead nanometric field-effect transistor sensors for single-cell analysis. ACS Nano **10**, 3214–3221 (2016). 10.1021/acsnano.5b05211 26816294 10.1021/acsnano.5b05211 PMC4933202

[110] F. Torricelli, D.Z. Adrahtas, Z. Bao, M. Berggren, F. Biscarini et al., Electrolyte-gated transistors for enhanced performance bioelectronics. Nat. Rev. Meth. Primers **1**, 66 (2021). 10.1038/s43586-021-00065-8 10.1038/s43586-021-00065-8 PMC9037952 35475166

[111] D. Kireev, M. Brambach, S. Seyock, V. Maybeck, W. Fu et al., Graphene transistors for interfacing with cells: towards a deeper understanding of liquid gating and sensitivity. Sci. Rep. **7**, 6658 (2017). 10.1038/s41598-017-06906-5 28751775 10.1038/s41598-017-06906-5 PMC5532278

[112] L. Capua, S. Sheibani, S. Kamaei, J. Zhang, A.M. Ionescu, Extended-Gate FET cortisol sensor for stress disorders based on aptamers-decorated graphene electrode: fabrication, Experiments and Unified Analog Predictive Modeling. 2020 IEEE International Electron Devices Meeting (IEDM). San Francisco, CA, USA. IEEE, (2020), 35.2.1–35.2.4.

[113] S.J. Park, S.E. Seo, K.H. Kim, S.H. Lee, J. Kim et al., Real-time monitoring of geosmin based on an aptamer-conjugated graphene field-effect transistor. Biosens. Bioelectron. **174**, 112804 (2021). 10.1016/j.bios.2020.112804 33257183 10.1016/j.bios.2020.112804

[114] A.K. Geim, D. Jiang, E.H. Hill, F. Schedin, K.S. Novoselov et al., Detection of individual gas molecules absorbed on graphene. arXiv e-prints. (2006) 10.1038/nmat1967 17660825

[115] J. Ristein, W. Zhang, F. Speck, M. Ostler, L. Ley et al., Characteristics of solution gated field effect transistors on the basis of epitaxial graphene on silicon carbide. J. Phys. D Appl. Phys. **43**, 345303 (2010). 10.1088/0022-3727/43/34/345303

[116] Y. Ohno, K. Maehashi, Y. Yamashiro, K. Matsumoto, Electrolyte-gated graphene field-effect transistors for detecting pH and protein adsorption. Nano Lett. **9**, 3318–3322 (2009). 10.1021/nl901596m 19637913 10.1021/nl901596m

[117] C. Homma, M. Tsukiiwa, H. Noguchi, M. Tanaka, M. Okochi et al., Designable peptides on graphene field-effect transistors for selective detection of odor molecules. Biosens. Bioelectron. **224**, 115047 (2023). 10.1016/j.bios.2022.115047 36628827 10.1016/j.bios.2022.115047

[118] R. Negishi, H. Hirano, Y. Ohno, K. Maehashi, K. Matsumoto et al., Layer-by-layer growth of graphene layers on graphene substrates by chemical vapor deposition. Thin Solid Films **519**, 6447–6452 (2011). 10.1016/j.tsf.2011.04.229

[119] B.M. Blaschke, M. Lottner, S. Drieschner, A.B. Calia, K. Stoiber et al., Flexible graphene transistors for recording cell action potentials. 2D Mater. **3**, 025007 (2016). 10.1088/2053-1583/3/2/025007

[120] L.H. Hess, M. Jansen, V. Maybeck, M.V. Hauf, M. Seifert et al., Graphene transistor arrays for recording action potentials from electrogenic cells. Adv. Mater. **23**, 5045–5049, 4968 (2011). 10.1002/adma.201102990 10.1002/adma.201102990 21953832

[121] C. Xie, Z. Lin, L. Hanson, Y. Cui, B. Cui, Intracellular recording of action potentials by nanopillar electroporation. Nat. Nanotechnol. **7**, 185–190 (2012). 10.1038/nnano.2012.8 22327876 10.1038/nnano.2012.8 PMC3356686

[122] J. Abbott, T. Ye, D. Ham, H. Park, Optimizing nanoelectrode arrays for scalable intracellular electrophysiology. Acc. Chem. Res. **51**, 600–608 (2018). 10.1021/acs.accounts.7b00519 29437381 10.1021/acs.accounts.7b00519

[123] M. Dipalo, G. Melle, L. Lovato, A. Jacassi, F. Santoro et al., Plasmonic meta-electrodes allow intracellular recordings at network level on high-density CMOS-multi-electrode arrays. Nat. Nanotechnol. **13**, 965–971 (2018). 10.1038/s41565-018-0222-z 30104618 10.1038/s41565-018-0222-z

[124] M. Dipalo, H. Amin, L. Lovato, F. Moia, V. Caprettini et al., Intracellular and extracellular recording of spontaneous action potentials in mammalian neurons and cardiac cells with 3D plasmonic nanoelectrodes. Nano Lett. **17**, 3932–3939 (2017). 10.1021/acs.nanolett.7b01523 28534411 10.1021/acs.nanolett.7b01523 PMC5520104

[125] M. Dipalo, G.C. Messina, H. Amin, R. La Rocca, V. Shalabaeva et al., 3D plasmonic nanoantennas integrated with MEA biosensors. Nanoscale **7**, 3703–3711 (2015). 10.1039/c4nr05578k 25640283 10.1039/c4nr05578k

[126] E.A. Woodcock, S.J. Matkovich, Cardiomyocytes structure, function and associated pathologies. Int. J. Biochem. Cell Biol. **37**, 1746–1751 (2005). 10.1016/j.biocel.2005.04.011 15950518 10.1016/j.biocel.2005.04.011

[127] D.M. Bers, S. Despa, Cardiac excitation–contraction coupling. *Encyclopedia of Biological Chemistry*. Amsterdam: Elsevier, (2013), 379–383. 10.1016/b978-0-12-378630-2.00221-8

[128] T. Kuo, Peter, Cardiac electrophysiology: From cell to bedside. JAMA **274**(6), 507 (1991). 10.1001/jama.1995.03530060083043

[129] D. Später, E.M. Hansson, L. Zangi, K.R. Chien, How to make a cardiomyocyte. Development **141**, 4418–4431 (2014). 10.1242/dev.091538 25406392 10.1242/dev.091538

[130] A. Leri, M. Rota, F.S. Pasqualini, P. Goichberg, P. Anversa, Origin of cardiomyocytes in the adult heart. Circ. Res. **116**, 150–166 (2015). 10.1161/CIRCRESAHA.116.303595 25552694 10.1161/CIRCRESAHA.116.303595 PMC4283577

[131] L.F. Santana, E.P. Cheng, W.J. Lederer, How does the shape of the cardiac action potential control calcium signaling and contraction in the heart? J. Mol. Cell. Cardiol. **49**, 901–903 (2010). 10.1016/j.yjmcc.2010.09.005 20850450 10.1016/j.yjmcc.2010.09.005 PMC3623268

[132] E. Carmeliet, J. Vereecke, Adrenaline and the plateau phase of the cardiac action potential. Importance of Ca++, Na+ and K+ conductance. Pflugers Arch. **313**, 300–315 (1969). 10.1007/BF00593955 10.1007/BF00593955 4391401

[133] C.H. Luo, Y. Rudy, A model of the ventricular cardiac action potential. Depolarization, repolarization, and their interaction. Circ. Res. **68**, 1501–1526 (1991). 10.1161/01.res.68.6.1501 10.1161/01.res.68.6.1501 1709839

[134] Z.C. Lin, A.F. McGuire, P.W. Burridge, E. Matsa, H.Y. Lou et al., Accurate nanoelectrode recording of human pluripotent stem cell-derived cardiomyocytes for assaying drugs and modeling disease. Microsyst. Nanoeng. **3**, 16080 (2017). 10.1038/micronano.2016.80 31057850 10.1038/micronano.2016.80 PMC6444980

[135] Y. Liang, M. Ernst, F. Brings, D. Kireev, V. Maybeck et al., High performance flexible organic electrochemical transistors for monitoring cardiac action potential. Adv. Healthc. Mater. **7**, e1800304 (2018). 10.1002/adhm.201800304 30109770 10.1002/adhm.201800304

[136] S. Syama, P.V. Mohanan, Comprehensive application of graphene: emphasis on biomedical concerns. Nano-Micro Lett. **11**, 6 (2019). 10.1007/s40820-019-0237-5 10.1007/s40820-019-0237-5 PMC7770934 34137957

[137] L. Zhou, K. Wang, H. Sun, S. Zhao, X. Chen et al., Novel graphene biosensor based on the functionalization of multifunctional nano-bovine serum albumin for the highly sensitive detection of cancer biomarkers. Nano-Micro Lett. **11**, 20 (2019). 10.1007/s40820-019-0250-8 10.1007/s40820-019-0250-8 PMC7770693 34137997

[138] Z. Zhu, An overview of carbon nanotubes and graphene for biosensing applications. Nano-Micro Lett. **9**, 25 (2017). 10.1007/s40820-017-0128-6 10.1007/s40820-017-0128-6 PMC6199032 30393720

[139] D. Kireev, S. Seyock, J. Lewen, V. Maybeck, B. Wolfrum et al., Graphene multielectrode arrays as a versatile tool for extracellular measurements. Adv. Healthc. Mater. **6**, 1601433 (2017). 10.1002/adhm.201601433 10.1002/adhm.201601433 28371490

[140] P.D. Nguyen, F. Ding, S.A. Fischer, W. Liang, X. Li, Solvated first-principles excited-state charge-transfer dynamics with time-dependent polarizable continuum model and solvent dielectric relaxation. J. Phys. Chem. Lett. **3**, 2898–2904 (2012). 10.1021/jz301042f

[141] L.H. Hess, C. Becker-Freyseng, M.S. Wismer, B.M. Blaschke, M. Lottner et al., Electrical coupling between cells and graphene transistors. Small **11**, 1703–1710 (2015). 10.1002/smll.201402225 25408432 10.1002/smll.201402225

[142] F. Veliev, A. Cresti, D. Kalita, A. Bourrier, T. Belloir et al., Sensing ion channel in neuron networks with graphene field effect transistors. 2D Mater. **5**, 045020 (2018). 10.1088/2053-1583/aad78f

[143] J. Chen, D. Chen, Y. Xie, T. Yuan, X. Chen, Progress of microfluidics for biology and medicine. Nano-Micro Lett. **5**, 66–80 (2013). 10.1007/BF03354852

[144] V. Dupuit, O. Terral, G. Bres, A. Claudel, B. Fernandez et al., A multifunctional hybrid graphene and microfluidic platform to interface topological neuron networks. Adv. Funct. Mater. **32**, 2207001 (2022). 10.1002/adfm.202207001

[145] J.A. Huang, V. Caprettini, Y. Zhao, G. Melle, N. Maccaferri et al., On-demand intracellular delivery of single particles in single cells by 3D hollow nanoelectrodes. Nano Lett. **19**, 722–731 (2019). 10.1021/acs.nanolett.8b03764 30673248 10.1021/acs.nanolett.8b03764 PMC6378653

[146] V. Caprettini, J.A. Huang, F. Moia, A. Jacassi, C.A. Gonano et al., Enhanced Raman investigation of cell membrane and intracellular compounds by 3D plasmonic nanoelectrode arrays. Adv. Sci. **5**, 1800560 (2018). 10.1002/advs.201800560 10.1002/advs.201800560 PMC6299714 30581692

[147] M. Donnelly, D. Mao, J. Park, G. Xu, Graphene field-effect transistors: the road to bioelectronics. J. Phys. D Appl. Phys. **51**, 493001 (2018). 10.1088/1361-6463/aadcca

[148] D. Xu, Z. Hu, J. Su, F. Wu, W. Yuan, Micro and nanotechnology for intracellular delivery therapy protein. Nano-Micro Lett. **4**, 118–123 (2012). 10.1007/BF03353702

[149] L. Raes, S. Stremersch, J.C. Fraire, T. Brans, G. Goetgeluk et al., Intracellular delivery of mRNA in adherent and suspension cells by vapor nanobubble photoporation. Nano-Micro Lett. **12**, 185 (2020). 10.1007/s40820-020-00523-0 10.1007/s40820-020-00523-0 PMC7770675 34138203

[150] H. Yin, W. Jiang, Y. Liu, D. Zhang, F. Wu et al., Advanced near-infrared light approaches for neuroimaging and neuromodulation. BMEMat **1**, e12023 (2023). 10.1002/bmm2.12023

[151] C. Lin, X. Li, T. Wu, J. Xu, Z. Gong et al., Optofluidic identification of single microorganisms using fiber-optical-tweezer-based Raman spectroscopy with artificial neural network. BMEMat **1**, e12007 (2023). 10.1002/bmm2.12015

[152] H. Song, M. Kim, E. Kim, J. Lee, I. Jeong et al., Neuromodulation of the peripheral nervous system: Bioelectronic technology and prospective developments. BMEMat **1**, e12048 (2023). 10.1002/bmm2.12048

[153] Y. Wang, M.L. Adam, Y. Zhao, W. Zheng, L. Gao et al., Machine learning-enhanced flexible mechanical sensing. Nano-Micro Lett. **15**, 55 (2023). 10.1007/s40820-023-01013-9 10.1007/s40820-023-01013-9 PMC9936950 36800133

[154] B. Hou, X. Liu, Stretching boundaries in neurophysiological monitoring. BMEMat **1**, e12054 (2023). 10.1002/bmm2.12054

